# An On-Site InSAR Terrain Imaging Method with Unmanned Aerial Vehicles

**DOI:** 10.3390/s24072287

**Published:** 2024-04-03

**Authors:** Hsu-Yueh Chuang, Jean-Fu Kiang

**Affiliations:** Graduate Institute of Communication Engineering, National Taiwan University, Taipei 10617, Taiwan

**Keywords:** InSAR, unmanned aerial vehicle, digital elevation model (DEM), phase unwrapping, mean filter

## Abstract

An on-site InSAR imaging method carried out with unmanned aerial vehicles (UAVs) is proposed to monitor terrain changes with high spatial resolution, short revisit time, and high flexibility. To survey and explore a specific area of interest in real time, a combination of a least-square phase unwrapping technique and a mean filter for removing speckles is effective in reconstructing the terrain profile. The proposed method is validated by simulations on three scenarios scaled down from the high-resolution digital elevation models of the US geological survey (USGS) 3D elevation program (3DEP) datasets. The efficacy of the proposed method and the efficiency in CPU time are validated by comparing with several state-of-the-art techniques.

## 1. Introduction

Radars have been widely used for terrain surveillance under different weather conditions [1], which is crucial for environmental protection and natural disaster evaluation [2]. Synthetical aperture radars (SARs), including ALOS (L-band, 2006–2011) [3], Sentinel-1 (C-band, 2014–) [3], and UAVSAR (airborne, L-band and P-band, 2008–) [3], have been used for monitoring glaciers, volcanoes, earthquakes, and so on. TerraSAR-X, operating at X-band with 300 MHz bandwidth, offers spatial resolution of 0.6 m × 1.1 m (slant range × azimuth) in spotlight mode, 0.6 m × 0.24 m in staring spotlight mode, and 1.2 m × 3.3 m in stripmap mode [4,5].

The InSAR technique has been used for measuring surface topography and altimetry profile [6], mapping three-dimensional building shape [7], and detecting building edge [8]. InSAR and TomoSAR imaging techniques demand precise coregistration between master image and slave images [9,10]. In [9], a two-step, scale-invariant feature transform (SIFT) registration method was proposed. In [11], an outlier-detecting total least-squares (OD-TLS) algorithm was proposed to enhance the precision and robustness of 3D point-set registration. In [12], a sinc interpolation method was used to implement subpixel-to-subpixel match.

Faithful reconstruction of a terrain profile relies on accurate acquisition of interferometric phase. Numerous filtering methods on interferometric phase have been developed in the past few decades [13], including transform domain methods [14], nonlocal methods [15], and spatial domain methods [16]. The trade-off between noise reduction and preservation of terrain-related signal with transform domain methods is typically adjusted via a threshold [14].

In [17], a 3D space-time nonlocal mean filter (NLMF) was applied to detect terrain changes by extracting nonlocal information from pixels in SAR images acquired in different time windows. In [18], a nonlocal mean filter was applied to a few persistent scattering points in a search area to improve the accuracy of 3D deformation profile. The nonlocal filters performed well in preserving details of complex structures, but were less effective in removing speckle noise [15].

A spatial-domain Gaussian filter was used to reduce high-frequency noise while preserving deformation information [19]. It could reduce impulse noise and preserve edges by replacing each pixel with the mean value of its neighboring pixels [20], but the edges might become blurred due to loss of fine details. On the other hand, nonlocal filters preserve intricate details and adapt to local structures by considering pixel patch similarities, with the downside of computational complexity and sensitivity to parameters.

Phase unwrapping (PU) is a critical step to derive a faithful terrain profile from the interferometric phase of the acquired InSAR image, and the results are affected by the number of baselines used in probing the target area [21]. A phase unwrapping problem could be formulated as a wrap count classification task to invoke deep learning methods [22], as used in processing optical images [23,24]. In [25], a quality-guided algorithm was developed by unwrapping the phases along an optimal path in the interferometric phase image, based on a quality map of all edges in the image. Although the result is insensitive to noise, its performance relies on the quality map and the errors may propagate along the path.

A least-squares (LS) phase unwrapping method was formulated as a global optimization task [26], which may be sensitive to outliers and takes long computational time to process a large image. In [27], a phase unwrapping method was proposed by minimizing the difference between the discrete partial derivative of the wrapped phase function and that of the unwrapped phase function. The unwrapped phases were obtained by solving a Hunt’s matrix and a discrete Poisson’s equation, accelerated by using FFT, and the result was comparable to other methods.

InSAR imaging tasks have been operated on spaceborne [28], airborne [29], ground-based [30], and UAV-borne platforms [31]. Spaceborne platforms are typically used to survey wide areas or large-scale phenomena [4], airborne platforms are more flexible in path planning [32], and ground-based platforms are used to monitor local environment [33].

UAV-borne platforms [34,35,36] are expedient for monitoring local area of contingency and can achieve spatial resolution of 10 cm [37] in P and L bands [31]. For example, the Antarctic ice sheet (AIS) is covered with rifts and crevasses off the map, endangering the exploration personnel [38,39]. Satellite-borne sensors cannot provide updated images and information for on-site tasks [38,40], but can be complemented with the InSAR images acquired with UAVs. Typical satellite-borne platforms take days to revisit the same area, with a baseline of a few hundred meters, while UAV-borne platforms can revisit the same area immediately after the previous flight, with a baseline of a few meters.

The radar signals can be acquired in two separate flights with single-channel SAR or a single flight with dual-channel SAR [41]. Typical position accuracy of UAVs derived from GPS lies between 0.5 and 2 m [42], which can be enhanced to the centimeter level by using differential GPS (DGPS) technique [43] or real-time kinematic GPS [44]. The downside of deploying UAVs is the impact of airflow disturbance and platform perturbation [42], which can be mitigated by applying motion compensation and autofocusing techniques [45,46,47].

In this work, an on-site InSAR imaging method is proposed to reconstruct a high-resolution local terrain profile with UAV-borne SARs in L-band. A mean filter is used to reduce artifact speckles, and a least-squares phase unwrapping method is used to acquire 2D interferometric phase in almost real time. Three high-quality digital elevation models (DEMs) featuring volcano, glacier, and landslide, are retrieved from the US geological survey (USGS) 3D elevation program (3DEP) [48] to validate the efficacy of the proposed method. The performance is further evaluated by comparing the acquired InSAR images with their counterparts acquired using other state-of-the-art techniques under the effects of noise.

The rest of this paper is organized as follows: the proposed InSAR method is presented in Section 2, the simulation results are discussed in Section 3, and some conclusions are drawn in Section 4.

## 2. Proposed InSAR Method

**Figure 1:**
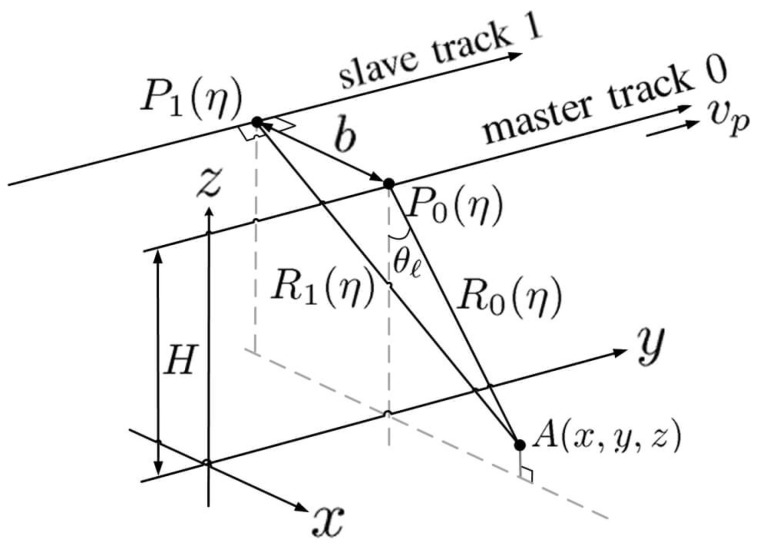
Schematic of InSAR operation.

Figure 1 shows the schematic of InSAR operation with two parallel flight paths, where the *x*, *y*, and *z* axes are aligned in the ground-range direction, azimuth direction, and height direction, respectively. A(x,y,z) denotes a point target, and the platform flies at height *H* above ground, with the side-looking angle $\theta_{\ell }$ to A(x,y,z).

The coordinates of radar $P_{0}\left(\eta \right)$ along the master track and radar $P_{1}\left(\eta \right)$ along the slave track are given by

(1) $$\begin{array}{ccc} & & \;P_{0}\left(\eta \right)=\left(0,\eta v_{p},H\right)\\ & & \;P_{1}\left(\eta \right)=\left(-b,\eta v_{p},H\right) \end{array}$$

The slant ranges from $P_{0}\left(\eta \right)$ and $P_{1}\left(\eta \right)$ to A(x,y,z) are $R_{0}\left(\eta \right)$ and $R_{1}\left(\eta \right)$, respectively, with

(2) $$\begin{array}{ccc} & & \;R_{0}\left(\eta \right)=\sqrt{{\left(x\right)}^{2}+{\left(y-\eta v_{p}\right)}^{2}+{\left(z-H\right)}^{2}}\\ & & \;R_{1}\left(\eta \right)=\sqrt{{\left(x+b\right)}^{2}+{\left(y-\eta v_{p}\right)}^{2}+{\left(z-H\right)}^{2}} \end{array}$$

### 2.1. Backscattered Signals

**Figure 2:**
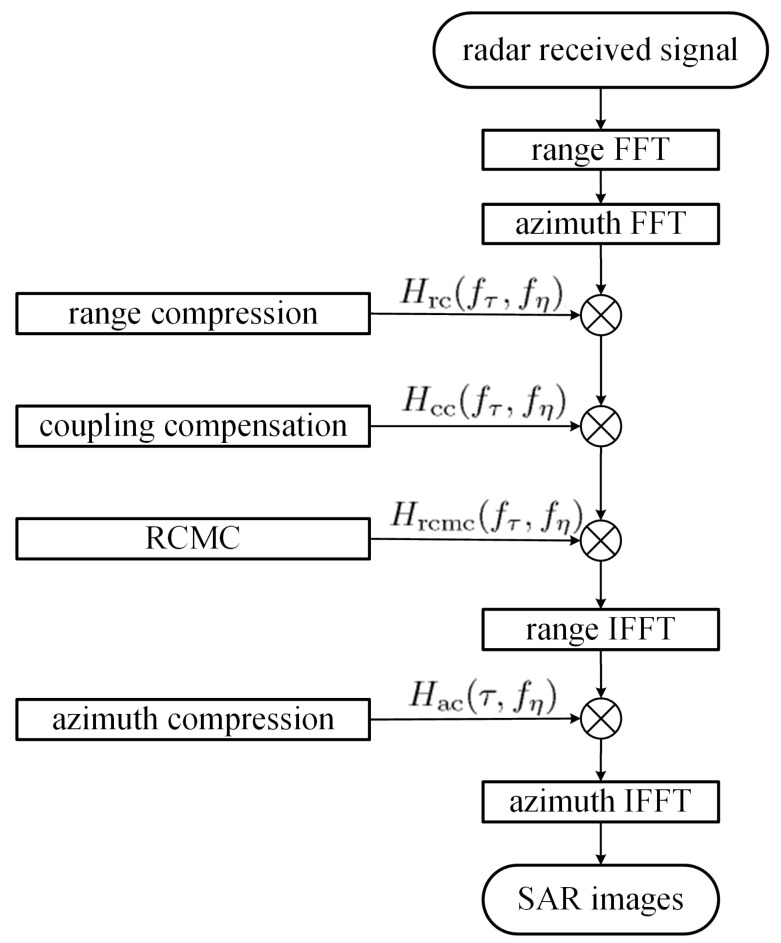
Flow-chart of range-Doppler algorithm (RDA) [49].

Figure 2 shows the flow-chart of the range-Doppler algorithm (RDA) used in this work [49]. The signal backscattered from the point target A(x,y,z) and received at $P_{n}\left(\eta \right)$ (n=0,1) is demodulated to the baseband as

(3) $$\begin{array}{ccc} & & \;s_{rn}\left(\tau ,\eta \right)=A_{0}w_{e}\left(\tau -2R_{n}\left(\eta \right)/c\right)e^{-j4\pi f_{0}R_{n}\left(\eta \right)/c+j\pi K_{r}{\left[\tau -2R_{n}\left(\eta \right)/c\right]}^{2}} \end{array}$$

where $A_{0}$ is the amplitude, $f_{0}$ is the carrier frequency, $K_{r}$ is the chirp rate of the linear frequency modulation (LFM) pulse, τ is the range (fast) time, η is the azimuth (slow) time, and $w_{e}\left(t\right)=\mathrm{rect}\left(t\right)$ is a window function, which is equal to one when |t|≤1/2 and zero otherwise.

By taking the Fourier transform of $s_{rn}\left(\tau ,\eta \right)$ with respect to τ and η sequentially, we have

(4) $$\begin{array}{ccc} & & \;S_{n}\left(f_{\tau },f_{\eta }\right)≃AW_{e}\left(f_{\tau }\right)e^{j\phi_{n}} \end{array}$$

where *A* is a constant of integration, $W_{e}\left(f_{\tau }\right)=w_{e}\left(f_{\tau }/K_{r}\right)$, and

(5) $$\begin{array}{ccc} & & \;\phi_{n}≃-\pi \frac{f_{\tau }^{2}}{K_{r}}-2\pi f_{\eta }\left(\frac{y}{v_{p}}\right)-\frac{4\pi R_{n}\left(0\right)D}{\lambda }-\frac{4\pi R_{n}\left(0\right)}{\lambda D}\frac{f_{\tau }}{f_{0}}\\ & & +\frac{4\pi R_{n}\left(0\right)}{\lambda }\frac{1-D^{2}}{2D^{3}}{\left(\frac{f_{\tau }}{f_{0}}\right)}^{2}-\frac{4\pi R_{n}\left(0\right)}{\lambda }\frac{1-D^{2}}{2D^{5}}{\left(\frac{f_{\tau }}{f_{0}}\right)}^{3} \end{array}$$

with $D=\sqrt{1-\frac{c^{2}f_{\eta }^{2}}{4f_{0}^{2}v_{p}^{2}}}$.

### 2.2. Range Compression

Let us define a range-compression filter $H_{\mathrm{rc}}\left(f_{\tau },f_{\eta }\right)$, a coupling-compensation filter $H_{\mathrm{cc}}\left(f_{\tau },f_{\eta }\right)$, and a range cell migration correction (RCMC) filter $H_{\mathrm{rcmc}}\left(f_{\tau },f_{\eta }\right)$ as

(6) $$\begin{array}{ccc} & & \;H_{\mathrm{rc}}\left(f_{\tau },f_{\eta }\right)=e^{j\pi f_{\tau }^{2}/K_{m}} \end{array}$$

(7) $$\begin{array}{ccc} & & \;H_{\mathrm{cc}}\left(f_{\tau },f_{\eta }\right)=\exp\left\{j\frac{\pi \lambda R_{n}\left(0\right)f_{\tau }^{3}f_{\eta }^{2}}{2D^{5}f_{0}^{3}v_{p}^{2}}\right\} \end{array}$$

(8) $$\begin{array}{ccc} & & \;H_{\mathrm{rcmc}}\left(f_{\tau },f_{\eta }\right)=\exp\left\{j\frac{4\pi R_{n}\left(0\right)f_{\tau }}{c}\left[\frac{1}{D}-\frac{1}{D(f_{\mathrm{dc}})}\right]\right\} \end{array}$$

where $f_{\mathrm{dc}}$ is the Doppler centroid and

(9) $$\begin{array}{ccc} & & \;\frac{1}{K_{m}}=\frac{1}{K_{r}}-\frac{4R_{n}\left(0\right)}{\lambda }\frac{1-D^{2}}{2D^{3}f_{0}^{2}} \end{array}$$

Then, multiply these three filters with $S_{n}\left(f_{\tau },f_{\eta }\right)$ to have

(10) $$\begin{array}{ccc} & & \;S_{n}^{\left(1\right)}\left(f_{\tau },f_{\eta }\right)=S_{n}\left(f_{\tau },f_{\eta }\right)H_{\mathrm{rc}}\left(f_{\tau },f_{\eta }\right)H_{\mathrm{cc}}\left(f_{\tau },f_{\eta }\right)H_{\mathrm{rcmc}}\left(f_{\tau },f_{\eta }\right)=AW_{e}\left(f_{\tau }\right)e^{j\phi_{n}^{\left(1\right)}} \end{array}$$

where

(11) $$\begin{array}{ccc} & & \;\phi_{n}^{\left(1\right)}=-2\pi f_{\eta }\left(\frac{y}{v_{p}}\right)-\frac{4\pi R_{n}\left(0\right)D}{\lambda }-\frac{4\pi R_{n}\left(0\right)}{\lambda D(f_{\mathrm{dc}})}\frac{f_{\tau }}{f_{0}} \end{array}$$

By taking the inverse Fourier transform of $S_{n}^{\left(1\right)}\left(f_{\tau },f_{\eta }\right)$ in the range, we obtain the range-compressed signal

(12) $$\begin{array}{ccc} & & \;S_{n}^{\left(2\right)}\left(\tau ,f_{\eta }\right)=Ae^{-j2\pi f_{\eta }y/v_{p}}e^{-j4\pi R_{n}\left(0\right)D/\lambda }K_{r}T_{r}\mathrm{sinc}\left\{K_{r}T_{r}\left[\tau -\frac{2R_{n}\left(0\right)}{cD(f_{\mathrm{dc}})}\right]\right\} \end{array}$$

where sinc(x)=sin(πx)/(πx).

### 2.3. Azimuth Compression

Let us define an azimuth compression filter

(13) $$\begin{array}{ccc} & & \;H_{\mathrm{ac}}\left(\tau ,f_{\eta }\right)=e^{j4\pi R_{n}\left(0\right)D/\lambda } \end{array}$$

which is multiplied with $S_{n}^{\left(2\right)}\left(\tau ,f_{\eta }\right)$ to have

(14) $$\begin{array}{ccc} & & \;S_{n}^{\left(3\right)}\left(\tau ,f_{\eta }\right)=S_{n}^{\left(2\right)}\left(\tau ,f_{\eta }\right)H_{\mathrm{ac}}\left(\tau ,f_{\eta }\right)\\ & & \;=AK_{r}T_{r}e^{-j2\pi f_{\eta }y/v_{p}}\mathrm{sinc}\left\{\left[\tau -\frac{2R_{n}\left(0\right)}{cD(f_{\mathrm{dc}})}\right]\right\} \end{array}$$

By taking the inverse Fourier transform of $S_{n}^{\left(3\right)}\left(\tau ,f_{\eta }\right)$ in azimuth, we obtain the azimuth-compressed signal

(15) $$\begin{array}{ccc} & & \;s_{n}^{\left(4\right)}\left(\tau ,\eta \right)=F_{\eta }^{-1}\left\{S_{n}^{\left(3\right)}\left(\tau ,f_{\eta }\right)\right\}\\ & & \;=AK_{r}T_{r}\mathrm{sinc}\left\{K_{r}T_{r}\left[\tau -\frac{2R_{n}\left(0\right)}{cD(f_{\mathrm{dc}})}\right]\right\}F_{a}\mathrm{sinc}\left\{F_{a}\left(\eta -y/v_{p}\right)\right\} \end{array}$$

which is the SAR image stored in a matrix $s_{n}^{\left(4\right)}\left[u,v\right]=s_{n}^{\left(4\right)}\left(\tau_{v},\eta_{u}\right)$ of size $N_{a}\times N_{r}$.

### 2.4. Coregistration

**Figure 3:**
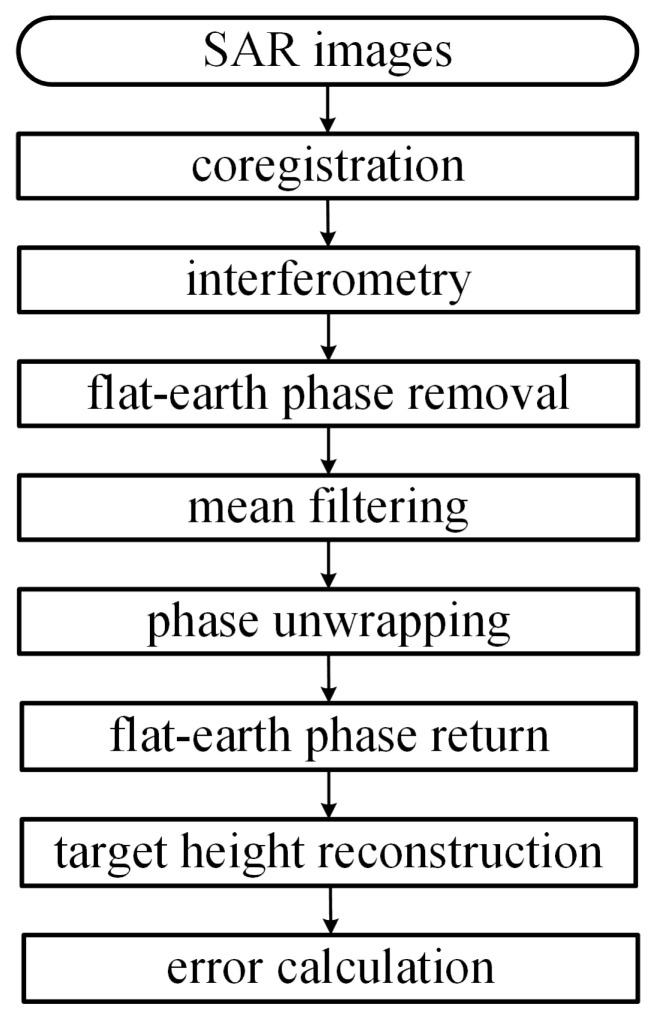
Flow-chart of InSAR imaging.

Figure 3 shows the flow-chart of InSAR imaging. In the master image, the τ-axis is sampled at $\tau_{a}+n_{r}\Delta \tau$, with $\tau_{a}=2R_{0}/c$ and $-N_{r}/2\leq n_{r}\leq N_{r}/2-1$. These sampling values of τ are stored in a vector

(16) $$\begin{array}{ccc} & & \;\bar{a}={\left[\tau_{a},\tau_{a},\cdots ,\tau_{a}\right]}^{t}+\Delta \tau {\left[-N_{r}/2,-N_{r}/2+1,\cdots ,N_{r}/2-1\right]}^{t} \end{array}$$

The slant ranges associated with all the range cells in the master image are ${\bar{r}}_{0}=c\bar{a}/2$, and the side-looking angle of the *v*th range cell is $\theta_{\ell }\left[v\right]=\cos^{-1}\left(H/r_{0}\left[v\right]\right)$, with $1\leq v\leq N_{r}$.

**Figure 4:**
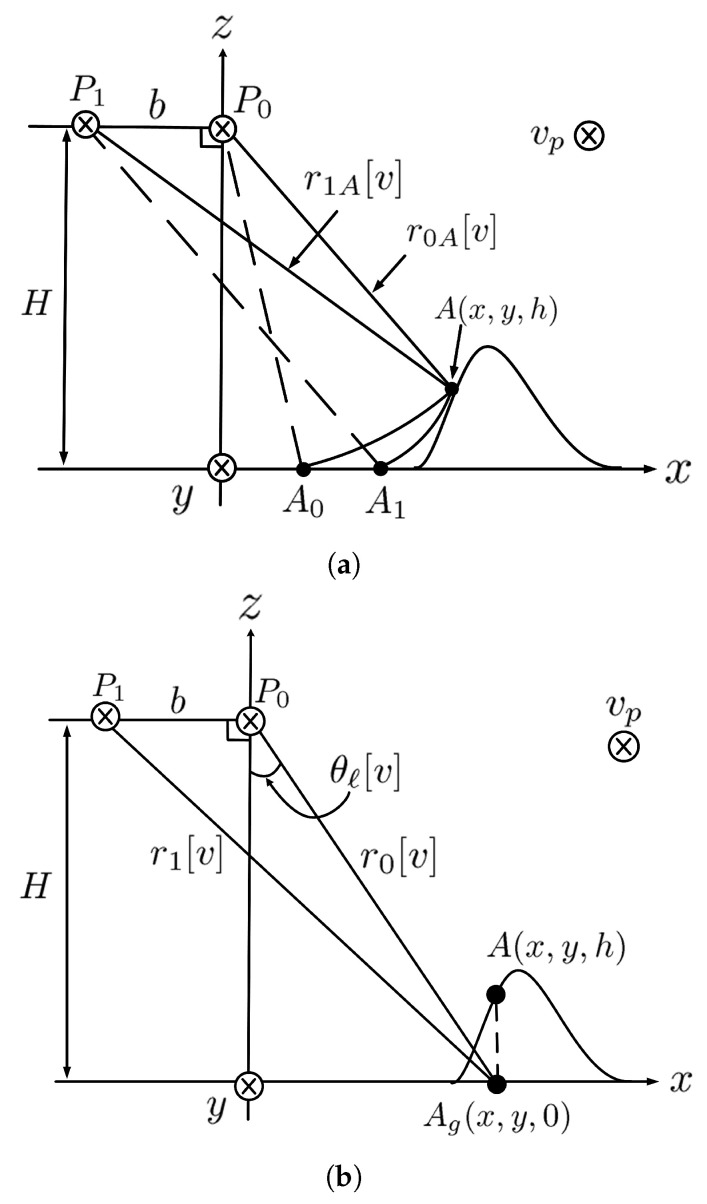
(**a**) Point target A(x,y,h) appears at $A_{0}$ in the master image and $A_{1}$ in the slave image. (**b**) Point target $A_{g}\left(x,y,0\right)$ with known $r_{0}\left[v\right]$, $r_{1}\left[v\right]$, $\theta_{\ell }\left[v\right]$, and *b*.

Figure 4a shows that the point target A(x,y,h) appears at $A_{0}$ in the master image and $A_{1}$ in the slave image. If the platforms fly high enough, the range difference between the two tracks in Figure 4a can be approximated as that in Figure 4b, namely, $\Delta r_{A}\left[v\right]=r_{1A}\left[v\right]-r_{0A}\left[v\right]≃r_{1}\left[v\right]-r_{0}\left[v\right]=\Delta r\left[v\right]$. By the law of cosines, $r_{1}\left[v\right]$ can be represented as $r_{1}\left[v\right]=\sqrt{b^{2}+{\left(r_{0}\left[v\right]\right)}^{2}-2br_{0}\left[v\right]\cos\left(\theta_{0}\left[v\right]+\pi /2\right)}$. The range difference Δr[v] is normalized with respect to $c/(2F_{r})$ to have $\Delta r_{p}\left[v\right]=\Delta r\left[v\right]\left(2F_{r}/c\right)$.

Next, apply both sinc interpolation [12] and subpixel-to-subpixel match to coregister the slave image to the master image. The original slave image ${\bar{\bar{s}}}_{1}^{\left(10\right)}$ of size $N_{a}\times N_{r}$ is interpolated in the range direction by a factor of 16 to obtain a finer slave image ${\bar{\bar{s}}}_{1}^{\left(13\right)}$ of size $N_{a}\times 16N_{r}$, which is resampled to derive a coregistered slave image $S_{1c}\left[u,v\right]$ of size $N_{a}\times N_{r}$.

### 2.5. Interferometry and Flat-Earth Phase Removal

An interferogram is formed from the master image $S_{0}\left[u,v\right]$ and the coregistered slave image $S_{1c}\left[u,v\right]$ as

(17) $$\begin{array}{ccc} & & \;I\left[u,v\right]=\;|S_{0}\left[u,v\right]||S_{1c}\left[u,v\right]|e^{j\phi [u,v]} \end{array}$$

where $\phi \left[u,v\right]=\phi_{0}\left[u,v\right]-\phi_{1c}\left[u,v\right]$ is the interferometric phase.

The interferometric phase attributed to the flat-earth reference plane is $\phi_{f}\left[v\right]=4\pi \left(r_{1}\left[v\right]-r_{0}\left[v\right]\right)/\lambda$ [50], which is subtracted from the phase of I[u,v] in (17) to obtain

(18) $$\begin{array}{ccc} & & \;I^{\left(1\right)}\left[u,v\right]=I\left[u,v\right]e^{-j\phi_{f}\left[v\right]}=\;|S_{0}\left[u,v\right]||S_{1c}\left[u,v\right]|e^{j\phi^{\left(1\right)}\left[u,v\right]} \end{array}$$

where $\phi^{\left(1\right)}\left[u,v\right]=\phi \left[u,v\right]-\phi_{f}\left[v\right]$.

### 2.6. Mean Filter

Since the master image and the slave image are not perfectly coregistered, the interferometric phase manifests some random noise, inflicting errors in the subsequent phase unwrapping process. A mean filter is applied before phase unwrapping to reduce such phase noise.

Consider a target area of $\left(2L_{a}+1\right)$ azimuth cells by $\left(2L_{r}+1\right)$ range cells, centered at $\left[N_{a}/2,N_{r}/2\right]$. The interferometric phase in the target area is mapped from $\phi^{\left(1\right)}\left[u,v\right]$ as

(19) $$\begin{array}{ccc} & & \;{\bar{\bar{\phi }}}^{\left(2\right)}=\left[\begin{array}{ccc} \phi^{\left(1\right)}\left[N_{a}/2+L_{a},N_{r}/2-L_{r}\right] & \cdots & \phi^{\left(1\right)}\left[N_{a}/2+L_{a},N_{r}/2+L_{r}\right]\\ \vdots & \ddots & \vdots \\ \phi^{\left(1\right)}\left[N_{a}/2-L_{a},N_{r}/2-L_{r}\right] & \cdots & \phi^{\left(1\right)}\left[N_{a}/2-L_{a},N_{r}/2+L_{r}\right] \end{array}\right] \end{array}$$

Next, apply a searching window of size $\left(2w_{a}+1\right)\times \left(2w_{r}+1\right)$ and centered at $\left[u^{\prime },v^{\prime }\right]$ on ${\bar{\bar{\phi }}}^{\left(2\right)}$ to have

$$\begin{array}{ccc} & & \;{\bar{\bar{\phi }}}_{u^{\prime }v^{\prime }}^{\left(3\right)}=\left[\begin{array}{ccc} \phi^{\left(2\right)}\left[u^{\prime }+w_{a},v^{\prime }-w_{r}\right] & \cdots & \phi^{\left(2\right)}\left[u^{\prime }+w_{a},v^{\prime }+w_{r}\right]\\ \vdots & \ddots & \vdots \\ \phi^{\left(2\right)}\left[u^{\prime }-w_{a},v^{\prime }-w_{r}\right] & \cdots & \phi^{\left(2\right)}\left[u^{\prime }-w_{a},v^{\prime }+w_{r}\right] \end{array}\right] \end{array}$$

An intermediate phase, $\phi_{s}\left[u^{\prime },v^{\prime }\right]$, is derived from ${\bar{\bar{\phi }}}_{u^{\prime }v^{\prime }}^{\left(3\right)}$ as [51]

(20) $$\begin{array}{ccc} & & \;A_{\phi_{s}}\left[u^{\prime },v^{\prime }\right]e^{j\phi_{s}\left[u^{\prime },v^{\prime }\right]}=\sum_{u^{\prime \prime }=u_{\min}^{\prime }}^{u_{\max}^{\prime }}\sum_{v^{\prime \prime }=v_{\min}^{\prime }}^{v_{\max}^{\prime }}e^{j\phi_{u^{\prime }v^{\prime }}^{\left(3\right)}\left[u^{\prime \prime },v^{\prime \prime }\right]} \end{array}$$

The interferometric phase after mean filtering is computed as [20]

$$\begin{array}{ccc} & & \;\phi^{\left(3^{\prime }\right)}\left[u^{\prime },v^{\prime }\right]=\phi_{s}\left[u^{\prime },v^{\prime }\right]+\frac{\phi^{\left(3\right)}\left[u^{\prime },v^{\prime }\right]-\phi_{s}\left[u^{\prime },v^{\prime }\right]}{\sum_{u^{\prime \prime }=u_{\min}^{\prime }}^{u_{\max}^{\prime }}\sum_{v^{\prime \prime }=v_{\min}^{\prime }}^{v_{\max}^{\prime }}\left\{\phi^{\left(3\right)}\left[u^{\prime \prime },v^{\prime \prime }\right]-\phi_{s}\left[u^{\prime },v^{\prime }\right]\right\}} \end{array}$$

### 2.7. Poisson’s Equation of Unwrapped Phase

Let us define a wrapping operator as [25]

$$\begin{array}{ccc} & & \;\phi^{\left(4\right)}\left[u^{\prime },v^{\prime }\right]=W\left\{\phi^{\left(3^{\prime }\right)}\left[u^{\prime },v^{\prime }\right]\right\}=\phi^{\left(3^{\prime }\right)}\left[u^{\prime },v^{\prime }\right]-2\pi \left\lfloor \frac{\phi^{\left(3^{\prime }\right)}\left[u^{\prime },v^{\prime }\right]+\pi }{2\pi }\right\rfloor \end{array}$$

which returns the principal value of $\phi^{\left(3^{\prime }\right)}\left[u^{\prime },v^{\prime }\right]$ in (−π,π]. The residue of $\phi^{\left(3^{\prime }\right)}\left[u^{\prime },v^{\prime }\right]$ is determined as [25]

(21) $$\begin{array}{ccc} & & \;R\left\{\phi^{\left(3^{\prime }\right)}\left[u^{\prime },v^{\prime }\right]\right\}=W\left\{\phi^{\left(3^{\prime }\right)}\left[u^{\prime },v^{\prime }\right]-\phi^{\left(3^{\prime }\right)}\left[u^{\prime }+1,v^{\prime }\right]\right\}\\ & & +W\left\{\phi^{\left(3^{\prime }\right)}\left[u^{\prime }+1,v^{\prime }\right]-\phi^{\left(3^{\prime }\right)}\left[u^{\prime }+1,v^{\prime }+1\right]\right\}\\ & & +W\left\{\phi^{\left(3^{\prime }\right)}\left[u^{\prime }+1,v^{\prime }+1\right]-\phi^{\left(3^{\prime }\right)}\left[u^{\prime },v^{\prime }+1\right]\right\}\\ & & +W\left\{\phi^{\left(3^{\prime }\right)}\left[u^{\prime },v^{\prime }+1\right]-\phi^{\left(3^{\prime }\right)}\left[u^{\prime },v^{\prime }\right]\right\} \end{array}$$

with possible outcomes of −2π,0, or 2π.

Next, take the mirror reflections of the wrapped phase function to obtain an even periodic function, which is continuous at the junction between two adjacent periods. Let $U=2L_{a}+1$ and $V=2L_{r}+1$, an expanded phase function is defined in terms of ${\bar{\bar{\phi }}}^{\left(4\right)}$ and its three versions of mirror reflection as

(22) $$\begin{array}{ccc} & & \;{\bar{\bar{\phi }}}_{2U\times 2V}^{\left(4^{\prime }\right)}=\left[\begin{array}{cc} {\bar{\bar{\phi }}}^{\left(4b\right)} & {\bar{\bar{\phi }}}^{\left(4c\right)}\\ {\bar{\bar{\phi }}}^{\left(4\right)} & {\bar{\bar{\phi }}}^{\left(4a\right)} \end{array}\right] \end{array}$$

where

(23) $$\begin{array}{ccc} & & \;{\bar{\bar{\phi }}}^{\left(4a\right)}=\left[\begin{array}{ccc} \phi^{\left(4\right)}\left[U,V\right] & \cdots & \phi^{\left(4\right)}\left[U,1\right]\\ \vdots & \ddots & \vdots \\ \phi^{\left(4\right)}\left[1,V\right] & \cdots & \phi^{\left(4\right)}\left[1,1\right] \end{array}\right]\\ & & \;{\bar{\bar{\phi }}}^{\left(4b\right)}=\left[\begin{array}{ccc} \phi^{\left(4\right)}\left[1,1\right] & \cdots & \phi^{\left(4\right)}\left[1,V\right]\\ \vdots & \ddots & \vdots \\ \phi^{\left(4\right)}\left[U,1\right] & \cdots & \phi^{\left(4\right)}\left[U,V\right] \end{array}\right]\\ & & \;{\bar{\bar{\phi }}}^{\left(4c\right)}=\left[\begin{array}{ccc} \phi^{\left(4\right)}\left[1,V\right] & \cdots & \phi^{\left(4\right)}\left[1,1\right]\\ \vdots & \ddots & \vdots \\ \phi^{\left(4\right)}\left[U,V\right] & \cdots & \phi^{\left(4\right)}\left[U,1\right] \end{array}\right] \end{array}$$

The wrapped phase differences

(24) $$\begin{array}{ccc} & & \;\Delta \phi_{v^{\prime }}\left[u^{\prime },v^{\prime }\right]=W\left\{\phi^{\left(4^{\prime }\right)}\left[u^{\prime },v^{\prime }+1\right]-\phi^{\left(4^{\prime }\right)}\left[u^{\prime },v^{\prime }\right]\right\}\\ & & \;\Delta \phi_{u^{\prime }}\left[u^{\prime },v^{\prime }\right]=W\left\{\phi^{\left(4^{\prime }\right)}\left[u^{\prime }+1,v^{\prime }\right]-\phi^{\left(4^{\prime }\right)}\left[u^{\prime },v^{\prime }\right]\right\} \end{array}$$

fall in (−π,π].

Given the wrapped phase $\phi^{\left(4^{\prime }\right)}\left[u^{\prime },v^{\prime }\right]$, its unwrapped counterpart, $\tilde{\phi }\left[u^{\prime },v^{\prime }\right]$ satisfies

(25) $$\begin{array}{ccc} & & \;\tilde{\phi }\left[u^{\prime }+1,v^{\prime }\right]-\tilde{\phi }\left[u^{\prime },v^{\prime }\right]=\Delta \phi_{u^{\prime }}\left[u^{\prime },v^{\prime }\right],\\ & & \;\;\;\;1\leq u^{\prime }\leq 2U-1,\;1\leq v^{\prime }\leq 2V \end{array}$$

(26) $$\begin{array}{ccc} & & \;\tilde{\phi }\left[u^{\prime },v^{\prime }+1\right]-\tilde{\phi }\left[u^{\prime },v^{\prime }\right]=\Delta \phi_{v^{\prime }}\left[u^{\prime },v^{\prime }\right],\\ & & \;\;\;\;1\leq u^{\prime }\leq 2U,\;1\leq v^{\prime }\leq 2V-1 \end{array}$$

The least-squares solution of (25) and (26) can be obtained by minimizing the cost function [27,52]

(27) $$\begin{array}{ccc} & & \;C=\sum_{u^{\prime }=1}^{2U-1}\sum_{v^{\prime }=1}^{2V}{\left\{\tilde{\phi }\left[u^{\prime }+1,v^{\prime }\right]-\tilde{\phi }\left[u^{\prime },v^{\prime }\right]-\Delta \phi_{u^{\prime }}\left[u^{\prime },v^{\prime }\right]\right\}}^{2}\\ & & +\sum_{u^{\prime }=1}^{2U}\sum_{v^{\prime }=1}^{2V-1}{\left\{\tilde{\phi }\left[u^{\prime },v^{\prime }+1\right]-\tilde{\phi }\left[u^{\prime },v^{\prime }\right]-\Delta \phi_{v^{\prime }}\left[u^{\prime },v^{\prime }\right]\right\}}^{2} \end{array}$$

with the Hunt’s method to have [52]

$$\begin{array}{ccc} & & \;\tilde{\phi }\left[u^{\prime }+1,v^{\prime }\right]+\tilde{\phi }\left[u^{\prime }-1,v^{\prime }\right]+\tilde{\phi }\left[u^{\prime },v^{\prime }+1\right]+\tilde{\phi }\left[u^{\prime },v^{\prime }-1\right]-4\tilde{\phi }\left[u^{\prime },v^{\prime }\right]\\ & & \;=\Delta \phi_{u^{\prime }}\left[u^{\prime },v^{\prime }\right]-\Delta \phi_{u^{\prime }}\left[u^{\prime }-1,v^{\prime }\right]+\Delta \phi_{v^{\prime }}\left[u^{\prime },v^{\prime }\right]-\Delta \phi_{v^{\prime }}\left[u^{\prime },v^{\prime }-1\right] \end{array}$$

which is rearranged into a Poisson’s difference equation on a 2U×2V grid as

(28) $$\begin{array}{ccc} & & \;\left\{\tilde{\phi }\left[u^{\prime }+1,v^{\prime }\right]-2\tilde{\phi }\left[u^{\prime },v^{\prime }\right]+\tilde{\phi }\left[u^{\prime }-1,v^{\prime }\right]\right\}\\ & & +\left\{\tilde{\phi }\left[u^{\prime },v^{\prime }+1\right]-2\tilde{\phi }\left[u^{\prime },v^{\prime }\right]+\tilde{\phi }\left[u^{\prime },v^{\prime }-1\right]\right\}=\rho \left[u^{\prime },v^{\prime }\right] \end{array}$$

where

(29) $$\begin{array}{ccc} & & \;\rho \left[u^{\prime },v^{\prime }\right]=\left(\Delta \phi_{u^{\prime }}\left[u^{\prime },v^{\prime }\right]-\Delta \phi_{u^{\prime }}\left[u^{\prime }-1,v^{\prime }\right]\right)+\left(\Delta \phi_{v^{\prime }}\left[u^{\prime },v^{\prime }\right]-\Delta \phi_{v^{\prime }}\left[u^{\prime },v^{\prime }-1\right]\right) \end{array}$$

### 2.8. Solving Poisson’s Difference Equation with FFT

Define the 2D discrete Fourier transform (DFT) of $\tilde{\phi }\left[u^{\prime },v^{\prime }\right]$ and its inverse as [52]

(30) $$\begin{array}{ccc} & & \;\Phi \left[m,n\right]=\sum_{u^{\prime }=1}^{2U}\sum_{v^{\prime }=1}^{2V}\tilde{\phi }\left[u^{\prime },v^{\prime }\right]\exp\left\{\frac{-j2\pi \left(m-1\right)\left(u^{\prime }-1\right)}{2U}\right\}\exp\left\{\frac{-j2\pi \left(n-1\right)\left(v^{\prime }-1\right)}{2V}\right\},\\ & & \;1\leq m\leq 2U,1\leq n\leq 2V \end{array}$$

(31) $$\begin{array}{ccc} & & \;\tilde{\phi }\left[u^{\prime },v^{\prime }\right]=\frac{1}{4UV}\sum_{m=1}^{2U}\sum_{n=1}^{2V}\Phi \left[m,n\right]\exp\left\{\frac{j2\pi \left(m-1\right)\left(u^{\prime }-1\right)}{2U}\right\}\exp\left\{\frac{j2\pi \left(n-1\right)\left(v^{\prime }-1\right)}{2V}\right\},\\ & & \;1\leq u^{\prime }\leq 2U,1\leq v^{\prime }\leq 2V \end{array}$$

By substituting (31) into the left-hand-side of (28), we obtain

(32) $$\begin{array}{ccc} & & \;\frac{1}{4UV}\sum_{m=1}^{2U}\sum_{n=1}^{2V}\Phi \left[m,n\right]e^{j\alpha }e^{j\beta }\{e^{j\pi (m-1)/U}+e^{-j\pi (m-1)/U}\\ & & +e^{j\pi (n-1)/V}+e^{-j\pi (n-1)/V}-4\} \end{array}$$

where $\alpha =\pi \left(m-1\right)\left(u^{\prime }-1\right)/U$ and $\beta =\pi \left(n-1\right)\left(v^{\prime }-1\right)/V$. The right-hand side of (28) can be represented as

(33) $$\begin{array}{ccc} & & \;\rho \left[u^{\prime },v^{\prime }\right]=\mathrm{IDFT}\left\{P\left[m,n\right]\right\}=\frac{1}{4UV}\sum_{m=1}^{2U}\sum_{n=1}^{2V}P\left[m,n\right]e^{j\alpha }e^{j\beta },\\ & & \;\;1\leq u^{\prime }\leq 2U,1\leq v^{\prime }\leq 2V \end{array}$$

By equating (32) and (33), we obtain

(34) $$\begin{array}{ccc} & & \;P\left[m,n\right]=\Phi \left[m,n\right]\left(2\cos\frac{\pi (m-1)}{U}+2\cos\frac{\pi (n-1)}{V}-4\right) \end{array}$$

The phase unwrapping procedure is summarized as follows:

**Step 1:** Take the mirror reflections of ${\bar{\bar{\phi }}}^{\left(4\right)}$ to obtain ${\bar{\bar{\phi }}}^{\left(4^{\prime }\right)}$, as in (22);

**Step 2:** Compute $\rho [u^{\prime },v^{\prime }]$ in (29), with $1\leq u^{\prime }\leq 2U$ and $1\leq v^{\prime }\leq 2V$;

**Step 3:** Take 2D DFT of $\rho [u^{\prime },v^{\prime }]$ to obtain P[m,n], as in (33);

**Step 4:** Compute Φ[m,n] by using (34), with 1≤m≤2U, 1≤n≤2V, and Φ[1,1]=0;

**Step 5:** Take 2D IDFT of Φ[m,n] to obtain the solution, $\tilde{\phi }\left[u^{\prime },v^{\prime }\right]$;

**Step 6:** Retrieve the unwrapped interferometric phases in the target area as

(35) $$\begin{array}{ccc} & & \;\phi^{\left(5\right)}\left[u^{\prime },v^{\prime }\right]=\left[\begin{array}{ccc} \tilde{\phi }\left[U,V\right] & \cdots & \tilde{\phi }\left[U,V\right]\\ \vdots & \ddots & \vdots \\ \tilde{\phi }\left[1,1\right] & \cdots & \tilde{\phi }\left[1,V\right] \end{array}\right] \end{array}$$

### 2.9. Nonlocal Filter

A nonlocal filter can be applied to either the interferometric phase $\phi^{\left(1\right)}\left[u^{\prime },v^{\prime }\right]$ in (18) before phase unwrapping or $\phi^{\left(5\right)}\left[u^{\prime },v^{\prime }\right]$ in (35) after phase unwrapping. The output of the nonlocal filter to $\phi^{\left(1\right)}\left[u^{\prime },v^{\prime }\right]$ is computed as [15,53]

(36) $$\begin{array}{ccc} & & \;\phi_{\mathrm{NL}}^{\left(2\right)}\left[u,v\right]=\sum_{\left[u^{\prime },v^{\prime }\right]\in W_{\mathrm{se}}}W_{1}\left[u,v;u^{\prime },v^{\prime }\right]\phi^{\left(1\right)}\left[u^{\prime },v^{\prime }\right] \end{array}$$

where $W_{\mathrm{se}}$ is a search window and $W_{1}\left[u,v;u^{\prime },v^{\prime }\right]$ is a weighting coefficient that is determined by the difference of pixels between two similarity windows centered at [u,v] and $\left[u^{\prime },v^{\prime }\right]$. The weighting coefficient is large if the pixels in these two similarity windows match closely, and vice versa. The sum of all weighting coefficients over $W_{\mathrm{se}}$ is set to one.

In the literature, a nonlocal filter is applied before phase unwrapping to reduce noise, speckle, or other artifacts embedded in the wrapped flattened phase, aiming to acquire a more accurate unwrapped phase. A nonlocal filter applied after phase unwrapping aims to smooth the unwrapped phase, at the risk of inducing artifacts or errors to the latter. The simulation results in this work show that smoother interferometric phase distribution is acquired by applying a nonlocal filter before phase unwrapping than after it.

### 2.10. Quality-Guided Phase Unwrapping

A quality-guided phase unwrapping process is also used in this work for comparison. A quality map is defined over a window $W_{s}$ centered at [u,v] as [25]

(37) $$\begin{array}{ccc} & & \;Z\left[u,v\right]=\sqrt{\sum_{\left[u^{\prime },v^{\prime }\right]\in W_{s}}{\left(\Delta \phi_{u}\left[u^{\prime },v^{\prime }\right]-\langle \Delta \phi_{u}\left[u,v\right]\rangle \right)}^{2}}\\ & & +\sqrt{\sum_{\left[u^{\prime },v^{\prime }\right]\in W_{s}}{\left(\Delta \phi_{v}\left[u^{\prime },v^{\prime }\right]-\langle \Delta \phi_{v}\left[u,v\right]\rangle \right)}^{2}} \end{array}$$

where $\Delta \phi_{u}\left[u^{\prime },v^{\prime }\right]$ and $\Delta \phi_{v}\left[u^{\prime },v^{\prime }\right]$ are the partial derivatives of the wrapped phase in the *u* and *v* directions, respectively, and their mean values over the window $W_{s}$ are denoted as $\langle \Delta \phi_{u}\left[u,v\right]\rangle$ and $\langle \Delta \phi_{v}\left[u,v\right]\rangle$, respectively.

After computing the quality map over an image area of interest, the pixel with the highest quality-map value is denoted as $\left[u_{s},v_{s}\right]$. The phase unwrapping process begins with its four surrounding pixels, $\left[u_{s}\pm 1,v_{s}\right]$ and $\left[u_{s},v_{s}\pm 1\right]$, followed by the pixels surrounding them. The process is repeated until all the pixels in the image area are exhausted.

### 2.11. Target Height Estimation

By adding the flat-earth phases in the target area,

(38) $$\begin{array}{ccc} & & \;{\bar{\phi }}_{f}^{\left(2\right)}=\left[\begin{array}{c} \phi_{f}^{\left(2\right)}\left[1\right]\\ \phi_{f}^{\left(2\right)}\left[2\right]\\ \vdots \\ \phi_{f}^{\left(2\right)}\left[2L_{r}+1\right] \end{array}\right]=\left[\begin{array}{c} \phi_{f}\left[N_{r}/2-L_{r}\right]\\ \phi_{f}\left[N_{r}/2-L_{r}+1\right]\\ \vdots \\ \phi_{f}\left[N_{r}/2+L_{r}\right] \end{array}\right] \end{array}$$

back to the unwrapping phase, $\phi^{\left(5\right)}\left[u^{\prime },v^{\prime }\right]$, we have

(39) $$\begin{array}{ccc} & & \;\phi^{\left(6\right)}\left[u^{\prime },v^{\prime }\right]=\phi^{\left(5\right)}\left[u^{\prime },v^{\prime }\right]+\phi_{f}^{\left(2\right)}\left[v^{\prime }\right] \end{array}$$

Without loss of generality, choose cell [1,1] as the reference cell, with a reference phase $\phi_{\mathrm{ref}}=\phi^{\left(6\right)}\left[1,1\right]-\phi_{f}^{\left(2\right)}\left[1\right]$. The phase difference between the master image and the slave image is calibrated as

(40) $$\begin{array}{ccc} & & \;\phi^{\left(7\right)}\left[u^{\prime },v^{\prime }\right]=\phi^{\left(6\right)}\left[u^{\prime },v^{\prime }\right]-\phi_{\mathrm{ref}} \end{array}$$

**Figure 5:**
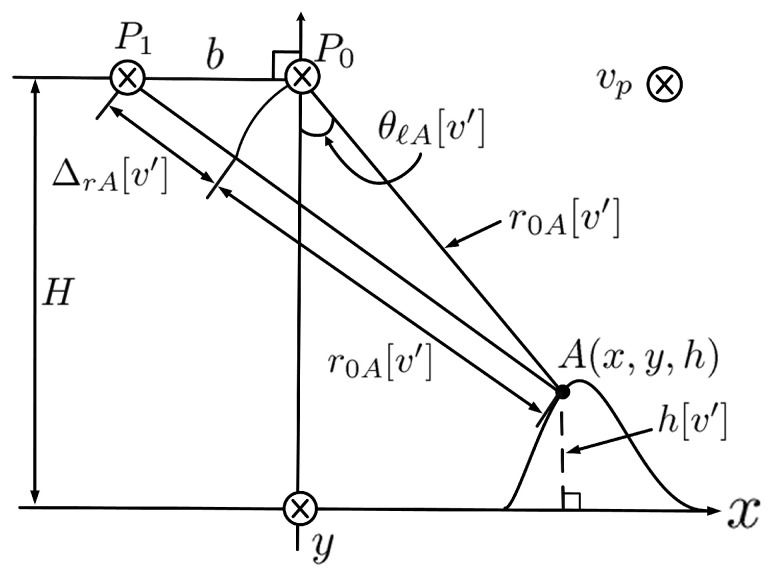
Geometry for target-height estimation.

Figure 5 shows the geometry for target-height estimation. The difference between $|\bar{P_{0}A}|$ and $|\bar{P_{1}A}|$ is estimated as

(41) $$\begin{array}{ccc} & & \;\Delta_{rA}\left[v^{\prime }\right]=\frac{\lambda }{4\pi }\phi^{\left(7\right)}\left[u^{\prime },v^{\prime }\right] \end{array}$$

The side-looking angle from the master track toward the point target *A* is calculated by using the law of cosines as

(42) $$\begin{array}{ccc} & & \;\theta_{\ell A}\left[v^{\prime }\right]=\cos^{-1}\left\{\frac{{\left(r_{0A}\left[v^{\prime }\right]\right)}^{2}+b^{2}-{\left(r_{0A}\left[v^{\prime }\right]+\Delta_{rA}\left[v^{\prime }\right]\right)}^{2}}{2br_{0A}\left[v^{\prime }\right]}\right\}-\frac{\pi }{2} \end{array}$$

Finally, the height of point target *A* is estimated as

(43) $$\begin{array}{ccc} & & \;h\left[v^{\prime }\right]=H-r_{0A}\left[u^{\prime },v^{\prime }\right]\cos\theta_{\ell A}\left[v^{\prime }\right] \end{array}$$

## 3. Simulations and Discussions

In this section, three scenarios are simulated by using the DEM models extracted from the US Geological Survey (USGS) 3D Elevation Program (3DEP) dataset [48], including Mount St. Helens, Columbia Glacier, and Santa Cruz landslide. Without loss of effectiveness, each DEM model is scaled down by a common factor in all three dimensions to reduce the computational time. Table 1 lists the default InSAR parameters used in the simulations, from which the height of ambiguity is determined as [54]

(44) $$\begin{array}{ccc} & & \;z_{\mathrm{amb}}=\frac{\lambda R_{0}\sin\theta_{\ell }}{2B_{⊥}}=80.52\;\left(m\right) \end{array}$$

Aside from the mean filter (MF) and the least-squares phase unwrapping (LSPU) method, the nonlocal filter (NF) and the quality-guided phase unwrapping (QGPU) method are also used for comparison. The effects of noise are studied by comparing the acquired images without noise with their counterparts at SNR =0 dB, −5 dB, and −10 dB.

### 3.1. Mount St. Helens

**Figure 6:**
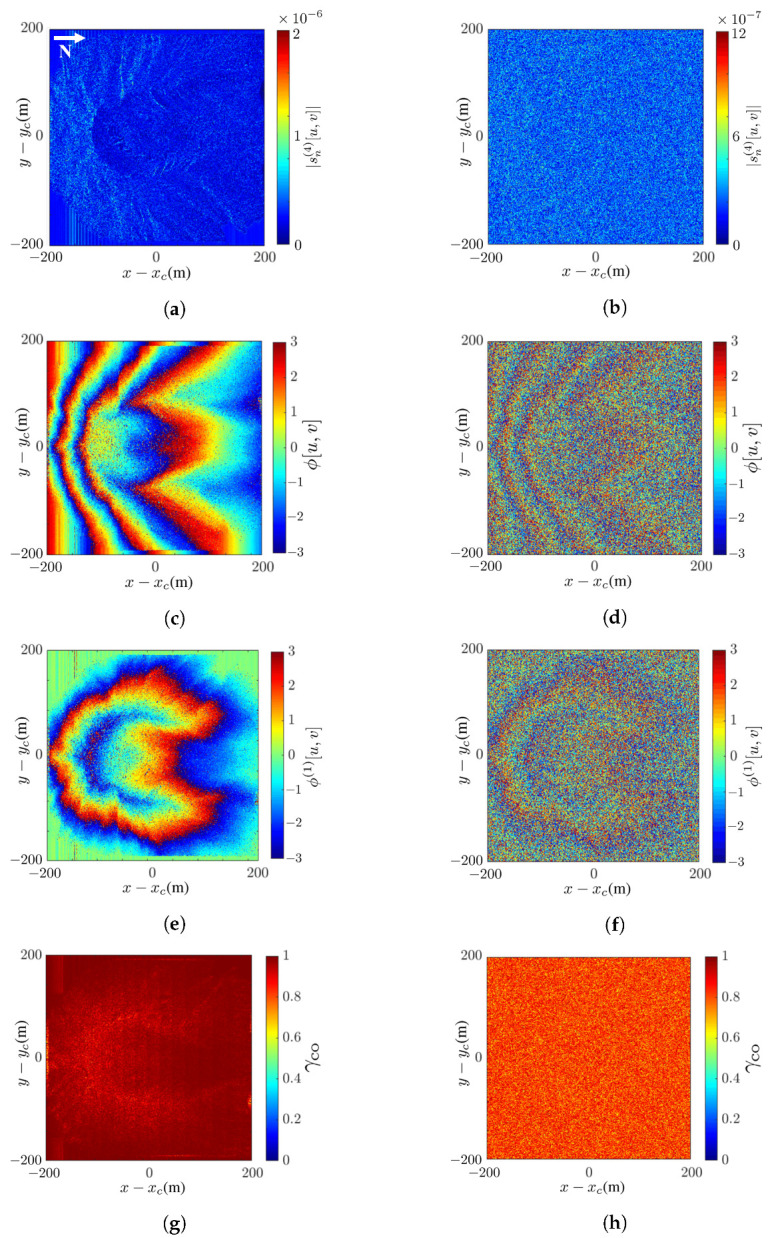
Intermediate images of Mount St. Helens: (**a**) master SAR image without noise, (**b**) master SAR image at SNR =−10 dB, (**c**) interferometric phase without noise, (**d**) interferometric phase at SNR =−10 dB, (**e**) wrapped flattened phase without noise, (**f**) wrapped flattened phase at SNR =−10 dB, (**g**) coherence map without noise, (**h**) coherence map at SNR =−10 dB.

Figure 6 shows the intermediate images of Mount St. Helens, scaled down tenfold to reduce the computational time. Mount St. Helens is an active volcano located at (46.2° N, 122.18° W), Skamania County, Washington, USA. Its elevation is 2549 m and its prominence is 1404 m. The DEM is extracted from the USGS 3DEP dataset [48], with spatial resolution of 1 m × 1 m.

Figure 6a,b shows the master SAR images without noise and at SNR =−10 dB, respectively. The latter manifests speckles over the whole image. Figure 6c,d shows the interferometric phase without noise and at SNR =−10 dB, respectively. The latter is severely smeared by noise and covered with speckles. Figure 6e,f shows the wrapped flattened phase without noise and at SNR =−10 dB, respectively. Similar features as in the interferograms are observed.

Figure 6g,h shows the coherence maps without noise and at SNR =−10 dB, respectively. The coherence between the master SAR image $S_{0}\left[u,v\right]$ and the coregistered slave image $S_{1c}\left[u,v\right]$ is defined as [54]

(45) $$\begin{array}{ccc} & & \;\gamma_{\mathrm{co}}\left[u,v\right]=\frac{E\{S_{0}\left[u,v\right]S_{1c}^{*}\left[u,v\right]\}}{\sqrt{E\{|S_{0}\left[u,v\right]{|}^{2}\}E\left\{|S_{1c}{\left[u,v\right]}^{2}\right\}}} \end{array}$$

which is equal to one if the coregistration is perfect. It is observed that the coherence map without noise is close to one, and that, at SNR =−10 dB, it is slightly reduced to about 0.8.

**Figure 7:**
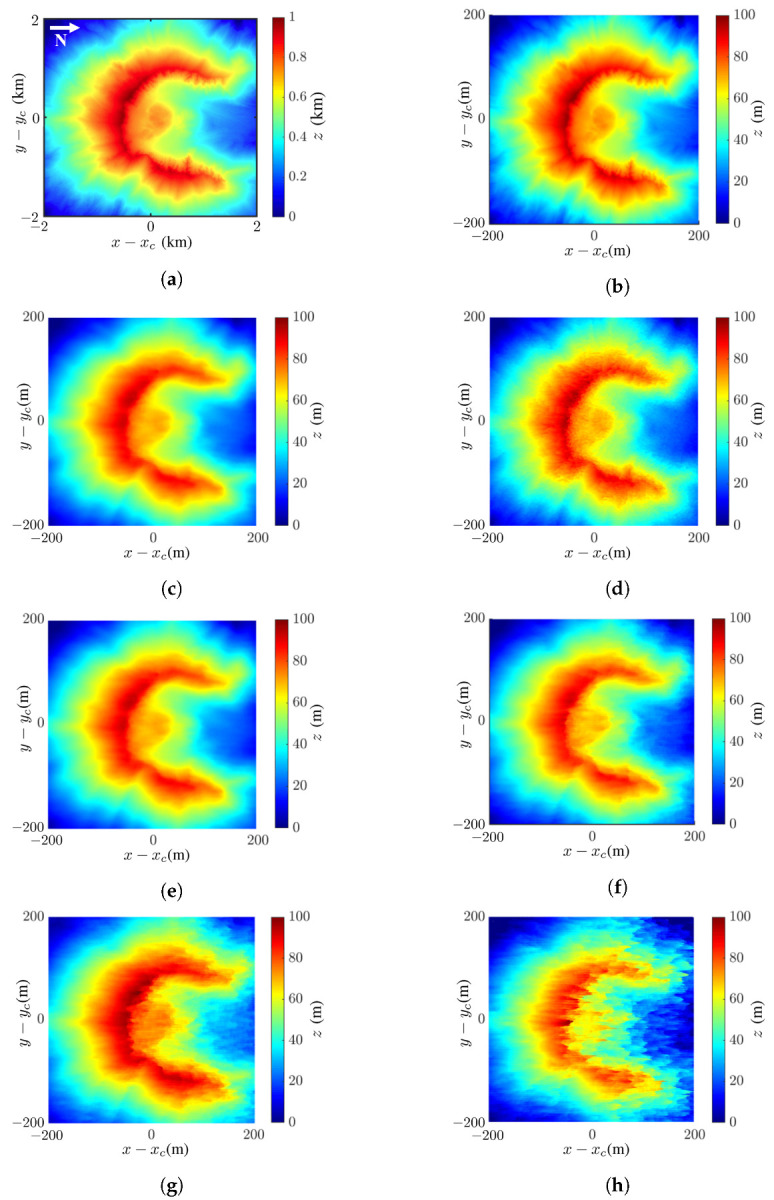
Images of Mount St. Helens: (**a**) DEM extracted from USGS 3DEP dataset [48], (**b**) tenfold scale-down model of DEM in (**a**); reconstructed DEM with (**c**) proposed method (MF and LSPU), SSIM =0.90, RMSE =5.79 m, (**d**) NF [15,53] and LSPU, SSIM =0.89, RMSE =6.14 m, (**e**) MF and QGPU [25], SSIM =0.90, RMSE =5.79 m, (**f**) proposed method at SNR =0 dB, SSIM =0.89, RMSE =10.8 m, (**g**) proposed method at SNR =−5 dB, SSIM =0.89, RMSE =9.22 m, (**h**) proposed method at SNR =−10 dB, SSIM =0.74, RMSE =22.38 m.

Figure 7 shows the reconstructed images of Mount St. Helens with the proposed method and the effects of noise. The comparison between mean filter (MF) and nonlocal filter (NF), as well as between least-squares phase unwrapping (LSPU) and quality-guided phase unwrapping (QGPU) methods, under noise free condition are also demonstrated.

Figure 7a shows the true DEM of Mount St. Helens extracted from the dataset, Figure 7b shows the tenfold scale-down model of that in Figure 7a, and Figure 7c shows the reconstructed DEM with the proposed method.

The fidelity of the acquired InSAR image *a* against the true image *b* is evaluated with a structural similarity (SSIM) index defined as [55,56]

(46) $$\begin{array}{ccc} & & \;\mathrm{SSIM}\left(a,b\right)=\left(\frac{2\mu_{a}\mu_{b}+c_{1}}{\mu_{a}^{2}+\mu_{b}^{2}+c_{1}}\right)\left(\frac{2\sigma_{ab}+c_{2}}{\sigma_{a}^{2}+\sigma_{b}^{2}+c_{2}}\right) \end{array}$$

where $\mu_{p}$ and $\sigma_{p}$ are the mean and standard deviation, respectively, of image *p*, with p=a,b; $\sigma_{ab}$ is the covariance between images *a* and *b*; and $c_{1}$ and $c_{2}$ are stability constants. The SSIM index lies in [0,1], with higher index indicating higher similarity. Each image pixel is stored in 8 bits, implying the dynamic range of $L=2^{8}-1=255$. The stability constants are chosen as $c_{1}={\left(0.01L\right)}^{2}=6.50$ and $c_{2}={\left(0.03L\right)}^{2}=58.52$. The SSIM index between the images in Figure 7b,c is 0.90.

The fidelity of the acquired InSAR image *a* against the true image *b* is also evaluated with a root-mean-square error (RMSE) defined as [57]

(47) $$\begin{array}{ccc} & & \;\mathrm{RMSE}\left(a,b\right)=\sqrt{\frac{1}{P}\sum_{p=1}^{P}{\left(a_{p}-b_{p}\right)}^{2}} \end{array}$$

where $a_{p}$ and $b_{p}$ are the values of the *p*th pixels in images *a* and *b*, respectively, and *P* is the number of pixels in one image. The RMSE between the images in Figure 7b,c is 5.79 m.

Figure 7d shows the reconstructed DEM, with the NF replacing the mean filter; its SSIM index and RMSE against the image in Figure 7b are 0.89 and 6.14 m, respectively, i.e., slightly worse than the proposed method.

A closer inspection of the images in Figure 7c,d reveals that the NF preserves sharper edge while the MF smears image features. The SSIM indices and RMSE values of these two images are similar, implying that MF and NF have comparable performance.

Figure 7e shows the reconstructed DEM with MF and QGPU; its SSIM index and RMSE against the image in Figure 7b are 0.90 and 5.79 m, respectively, which are identical to those in Figure 7c, indicating that LSPU and QGPU methods have comparable performance in this case. Note that the QGPU method has longer computation time than the LSPU method.

Table 2 lists the CPU time of running for LSPU, QGPU, mean filter, and nonlocal filter, with MATLAB R2019a on a PC with i7-3.00 GHz CPU and 32 GB memory. The CPU time of running for the mean filter is about half that of the nonlocal filter. The CPU time of the LSPU is much shorter than that of the QGPU because the former is implemented with FFT on the whole image, while the QGPU is executed pixel by pixel. The breakdown of CPU time in LSPU, QGPU, mean filter, and nonlocal filter, as well as their algorithms, are detailed in Appendix A.

Figure 7f–h shows the InSAR images acquired with the proposed method at SNR =0 dB, −5 dB, and −10 dB, respectively. Their SSIM indices against Figure 7b are 0.89, 0.89, and 0.74, respectively, and their RMSE values against Figure 7b are 10.8 m, 9.22 m, and 22.38 m, respectively. The main features in the image are almost unaffected at SNR =−5 dB and become slightly distorted at SNR =−10 dB. In short, the DEM of Mount St. Helens is reconstructed with high fidelity by visual inspection, as well as in terms of SSIM and RMSE, even at SNR =−10 dB.

**Figure 8:**
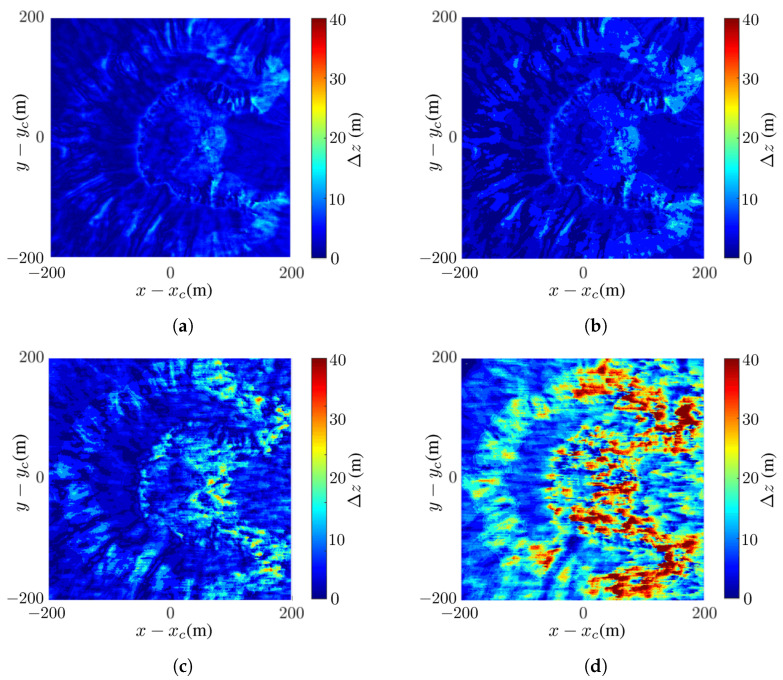
Difference between reconstructed DEM and true DEM in Figure 7b: (**a**) noise-free, (**b**) SNR =0 dB, (**c**) SNR =−5 dB, (**d**) SNR =−10 dB.

Figure 8 shows the differences between the reconstructed DEMs in Figure 7c,f,g,h and the true DEM in Figure 7b. The difference is calculated as $\Delta z=|a_{p}-b_{p}|$, where $a_{p}$ and $b_{p}$ are the values of the *p*th pixel in images *a* and *b*, respectively. Figure 8 shows that the difference is negligible at SNR ≤−5 dB and becomes significant at SNR =−10 dB.

**Figure 9:**
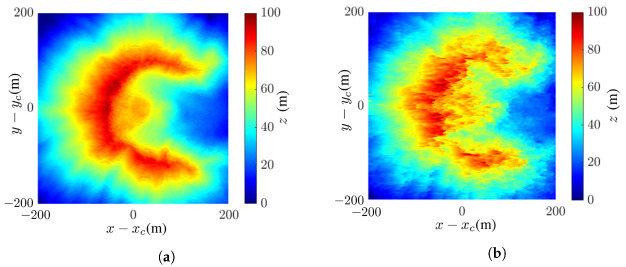
Reconstructed images of Mount St. Helens under noise-free condition: (**a**) with NF before phase unwrapping, (**b**) with NF after phase unwrapping.

Figure 9 shows the reconstructed images of Mount St. Helens, with the nonlocal filter (NF) applied before and after the LSPU, under noise-free condition. The computational noise distorts some terrain features and inflicts speckles in the reconstructed image if the nonlocal filter is applied after phase unwrapping.

### 3.2. Columbia Glacier

**Figure 10:**
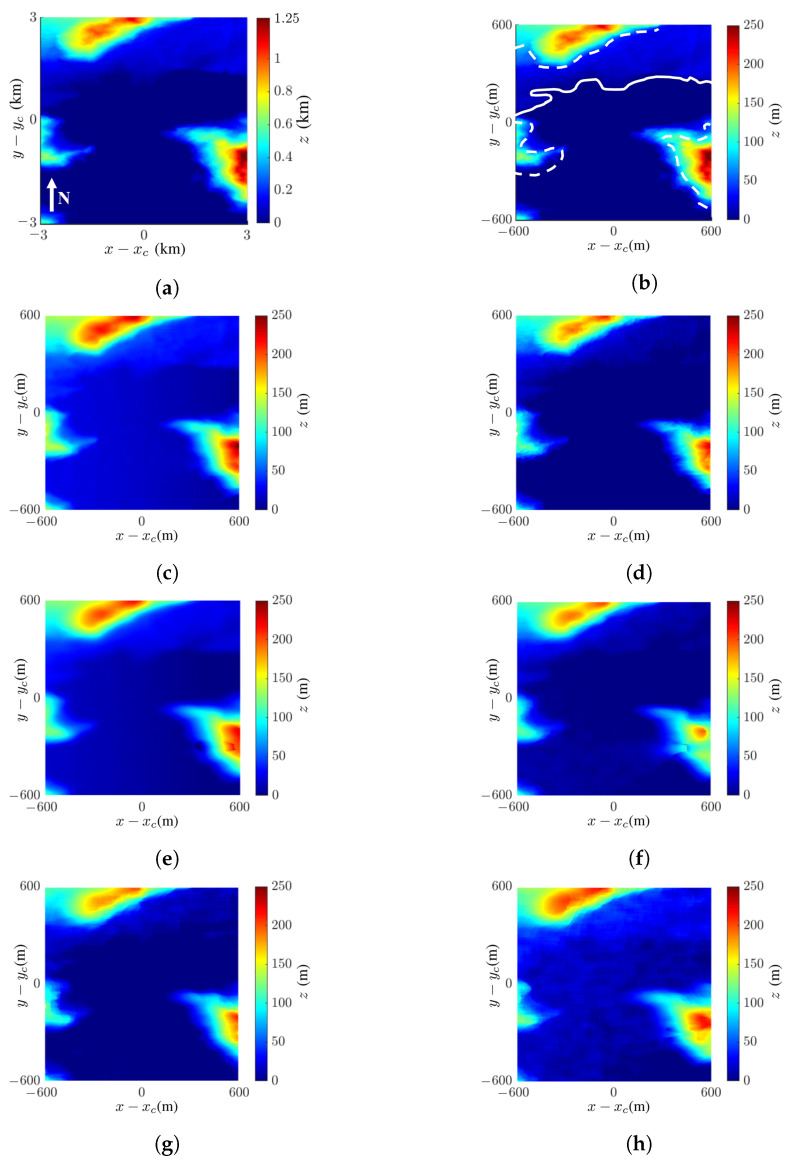
Images of Columbia Glacier: (**a**) DEM extracted from USGS 3DEP dataset [48], (**b**) fivefold scale-down model of (**a**)—glacier edge is marked by white curve, region of layover is marked by white dashed curve; reconstructed DEM with (**c**) proposed method, SSIM =0.88, RMSE =28.4 m, (**d**) NF and LSPU, SSIM =0.87, RMSE =28.24 m, (**e**) MF and QGPU, SSIM =0.88, RMSE =24.91 m, (**f**) proposed method at SNR =0 dB, SSIM =0.87, RMSE =31.93 m, (**g**) proposed method at SNR =−5 dB, SSIM =0.86, RMSE =30.24 m, (**h**) proposed method at SNR =−10 dB, SSIM =0.78, RMSE =33.24 m.

Figure 10 shows the images of the Columbia Glacier, located at (61.14° N, 147.08° W) on the south coast of Alaska, USA. The DEM is extracted from the USGS 3DEP dataset [48], with spatial resolution of 5 m × 5 m. Figure 10a shows the true DEM of the Columbia Glacier extracted from the dataset. Figure 10b shows the fivefold scale-down model of that in Figure 10a. Figure 10c shows the reconstructed DEM with the proposed method and the simulation parameters listed in Table 1. The reconstructed DEM closely matches the true DEM; its SSIM index and RMSE against the image in Figure 10b are 0.88 and 28.4 m, respectively.

**Figure 11:**
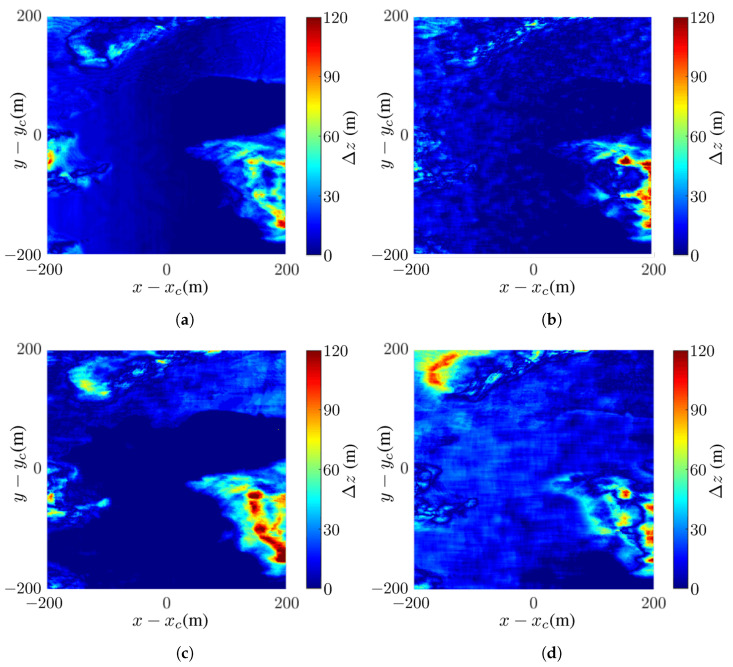
Difference between reconstructed DEM and true DEM in Figure 10b: (**a**) noise-free, (**b**) SNR =0 dB, (**c**) SNR =−5 dB, (**d**) SNR =−10 dB.

The backscattered signals from multiple resolution cells near the steep mountain slope region surrounding the glacier, enclosed by white dashed curves in Figure 10b, are mapped to the same resolution cell in the acquired image, inflicting layover effect. The high RMSE value is attributed to such layover regions, which is confirmed later in Figure 11.

Figure 10d shows the reconstructed DEM with NF replacing the mean filter; its SSIM index and RMSE against the image in Figure 10b are 0.87 and 28.24 m, respectively. Figure 10e shows the reconstructed DEM with QGPU replacing LSPU; its SSIM index and RMSE against the image in Figure 10b are 0.88 and 24.91 m, respectively, slightly better than their counterparts in Figure 10c. The glacier in this scenario manifests a steeper slope than that of the volcano in the previous scenario. The use of mean filter may blur some fine features in the DEM; hence, it should be used with caution if the terrain profile changes drastically.

Figure 10f–h shows the InSAR images acquired with the proposed method at SNR =0 dB, −5 dB, and −10 dB, respectively. Their SSIM indices against Figure 10b are 0.87, 0.86, and 0.78, respectively, and their RMSE values against Figure 10b are 31.93 m, 30.24 m, and 33.24 m, respectively. The acquired InSAR images at SNR =0 dB and SNR =−5 dB have similar SSIM indices, and the RMSE at SNR =−5 dB is slightly lower than the other two images.

Figure 11 shows the difference between the reconstructed DEM in Figure 10c,f–h, and the true DEM in Figure 10b. As SNR is decreased from 0 dB to −10 dB, more pixels in the layover regions manifest significant difference.

### 3.3. Santa Cruz Landslide

**Figure 12:**
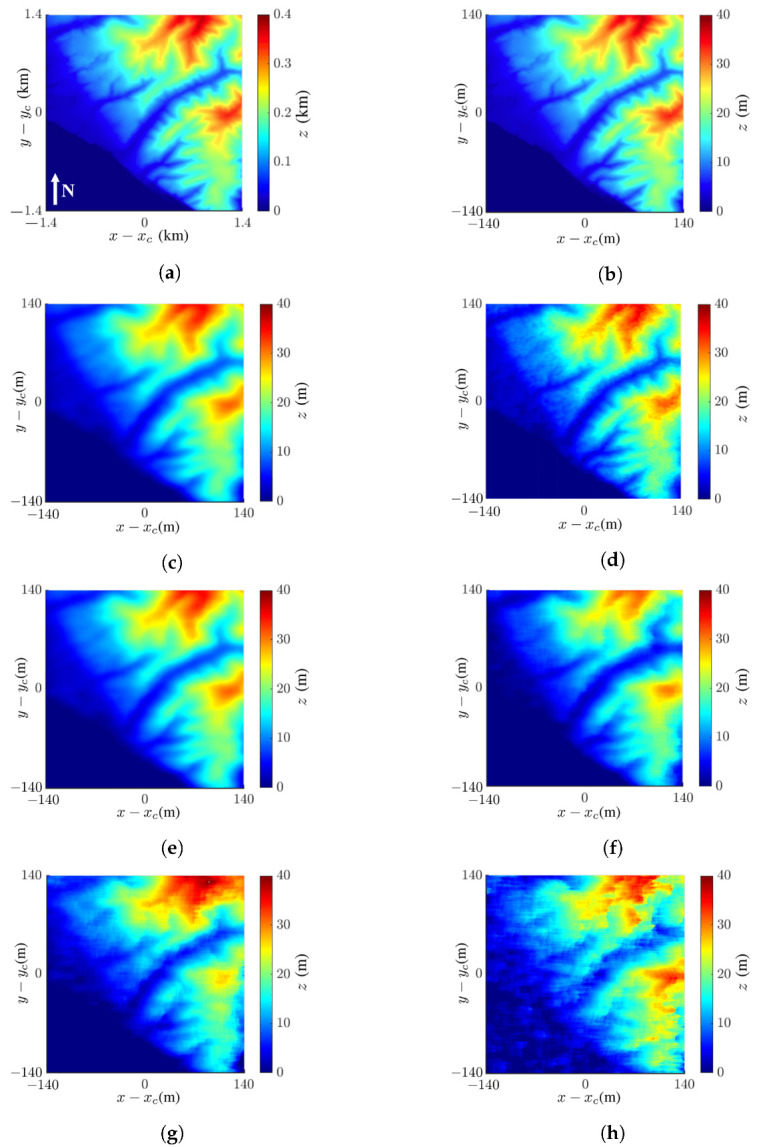
Images of landslide area near Santa Cruz on 17 March 2020: (**a**) DEM extracted from USGS 3DEP dataset [48], (**b**) tenfold scale-down model of (**a**); reconstructed DEM with (**c**) proposed method, SSIM =0.90, RMSE =2.32 m, (**d**) NF and LSPU, SSIM =0.89, RMSE =2.46 m, (**e**) MF and QGPU, SSIM =0.90, RMSE =2.32 m, (**f**) proposed method at SNR =0 dB, SSIM =0.90, RMSE =2.32 m, (**g**) proposed method at SNR =−5 dB, SSIM =0.72, RMSE =7.74 m, (**h**) proposed method at SNR =−10 dB, SSIM =0.66, RMSE =9.09 m.

Figure 12 shows the images of an area with potential landslide hazards near Santa Cruz (37.03° N, 122.12° W), California, USA, on 17 March 2020, which are extracted from the USGS 3DEP dataset [48], with spatial resolution of 3 m × 3 m. Figure 12a shows the true DEM of the target area, and Figure 12b shows the tenfold scale-down model of that in Figure 12a.

Figure 12c shows the reconstructed InSAR image with the proposed method. The reconstructed DEM closely matches the true DEM; its SSIM index and RMSE against the image in Figure 12b are 0.90 and 2.32 m, respectively. Figure 12d shows the reconstructed InSAR image, with the nonlocal filter replacing the mean filter. Its SSIM index and RMSE against the image in Figure 12b are 0.89 and 2.46 m, respectively. Figure 12e shows the reconstructed DEM, with QGPU replacing LSPU. Its SSIM index and RMSE against the DEM in Figure 12b are 0.90 and 2.32 m, respectively, same as those for the proposed method.

Figure 12f–h show the InSAR images acquired with the proposed method at SNR =0 dB, −5 dB, and −10 dB, respectively. Their SSIM indices against Figure 12b are 0.90, 0.72, and 0.66, respectively, and their RMSE values against Figure 12b are 2.32 m, 7.74 m, and 9.09 m, respectively.

**Figure 13:**
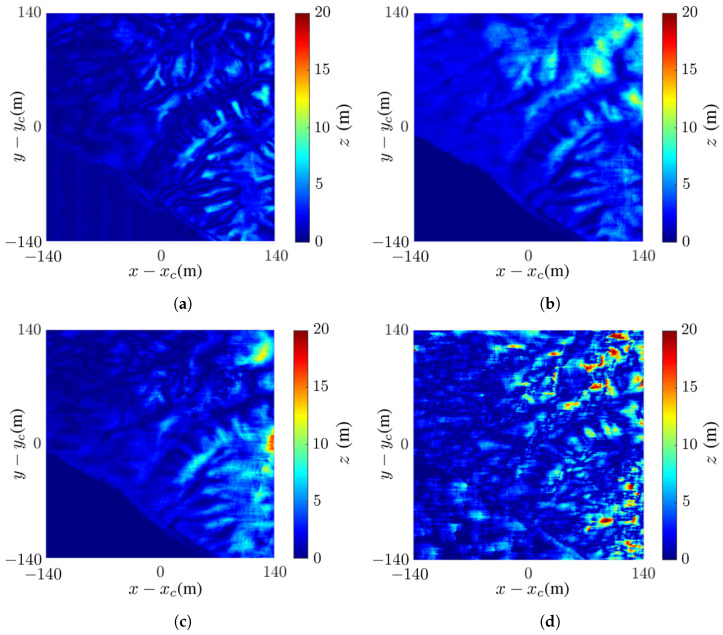
Difference between reconstructed DEM and true DEM in Figure 12b: (**a**) noise-free, (**b**) SNR =0 dB, (**c**) SNR =−5 dB, (**d**) SNR =−10 dB.

Figure 13 shows the differences between the reconstructed DEM in Figure 12c,f–h and the true DEM in Figure 12b. As SNR is decreased, more pixels manifest significant difference.

Table 3 summarizes the RMSE and SSIM indices of images in Figure 7, Figure 10, and Figure 12, with different combinations of the filter and phase unwrapping methods under noise-free condition. The best indices among the three different methods are marked by boldface, and the differences among these combinations are not significant.

Table 4 summarizes the RMSE and SSIM indices of images in Figure 7, Figure 10, and Figure 12, by using the proposed method under different SNRs. In general, the best indices occur at SNR =0 dB, but some indices at SNR =−5 dB turn out to be slightly better.

**Figure 14:**
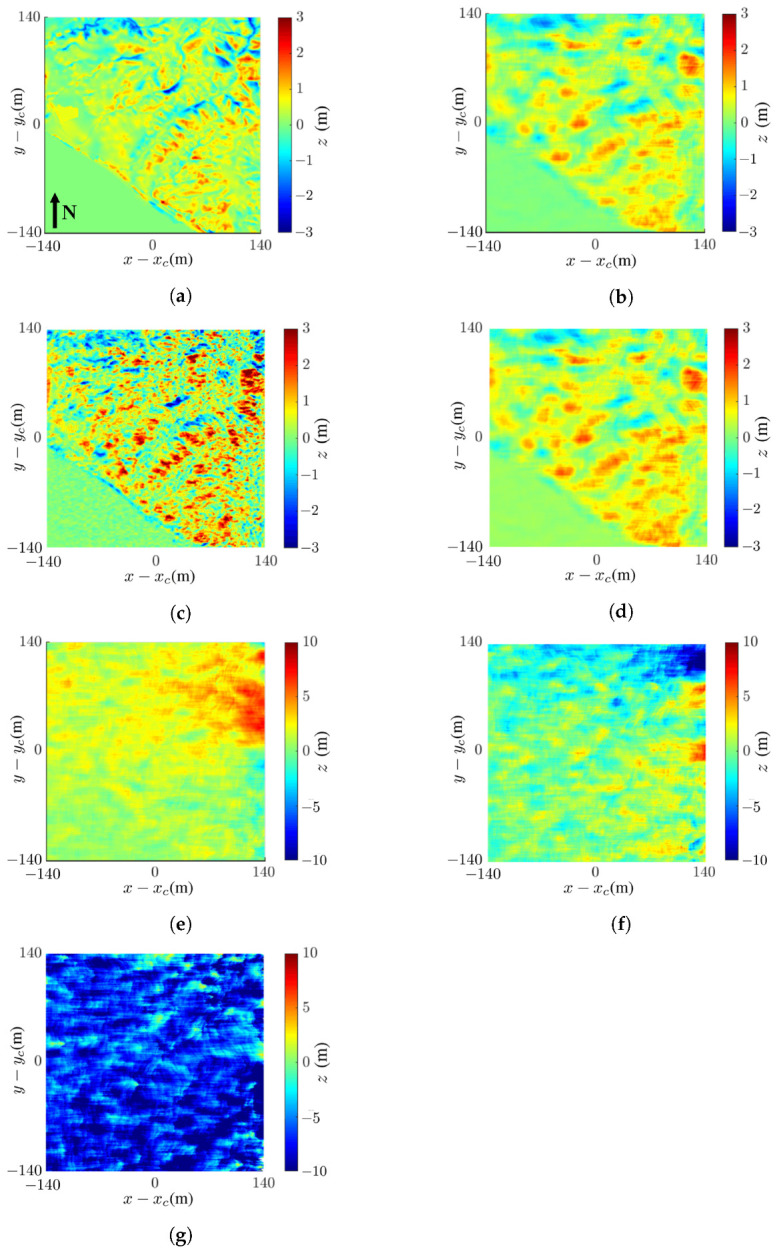
Height difference between 17 March 2020 and 10 August 2022 in landslide area near Santa Cruz,: (**a**) between DEM_*a*_ on 10 August 2022 and DEM_*b*_ in Figure 12b; reconstructed with (**b**) proposed method, SSIM =0.29, RMSE =2.32 m, (**c**) NF and LSPU, SSIM =0.30, RMSE =2.26 m, (**d**) MF and QGPU, SSIM =0.29, RMSE =2.26 m, (**e**) NF and LSPU at SNR =0 dB, SSIM =0.45, RMSE =4.31 m, (**f**) NF and LSPU at SNR =−5 dB, SSIM =0.20, RMSE =4.63 m, (**g**) NF and LSPU at SNR =−10 dB, SSIM =0.13, RMSE =9.54 m.

Next, we reconstruct two DEMs over the same area, dated 17 March 2020 and 10 August 2022, and show their height difference in Figure 14 to detect possible landslide hazards. Figure 14a shows the height difference between the two true DEMs extracted from the dataset on the two dates just mentioned [48]. Figure 14b shows the height difference between the two InSAR images reconstructed with the proposed method, and its SSIM index against the image in Figure 14a is 0.29. Both images show similar patterns, but some fine features in Figure 14a are smeared out in Figure 14b.

Figure 14c shows the height difference between the two images reconstructed with the nonlocal filter replacing the mean filter. The image shows a similar pattern as in Figure 14a, with more fragmented features than the latter. The SSIM index between these two images is 0.30.

Figure 14d shows the reconstructed image, with the QGPU replacing the LSPU. It is more resemblant of Figure 14b than Figure 14c, and its SSIM index against the image in Figure 14a is 0.29. By comparing Figure 14a–d, the combination of the NF and LSPU methods seems to manifest more terrain details in the true DEM.

Figure 14e–g shows the height differences acquired with the NF and LSPU at SNR =0 dB, −5 dB, and −10 dB, respectively. Their SSIM indices against Figure 14a are 0.45, 0.20, and 0.13, respectively, and their RMSE values against Figure 14a are 4.31 m, 4.63 m, and 9.54 m, respectively. The images in Figure 14e,f still retain some useful information about terrain profile change, but that in Figure 14g provides no useful clue.

**Figure 15:**
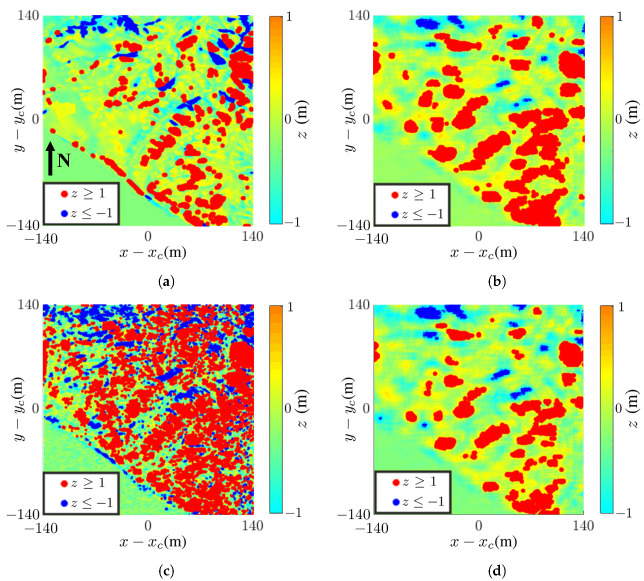
Density map of high-risk landslide areas derived from (**a**) Figure 14a, true DEM, (**b**) Figure 14b, proposed method, (**c**) Figure 14c, NF and LSPU, (**d**) Figure 14d, MF and QGPU.

Figure 15 shows the density maps of high-risk landslide areas acquired with the three methods compared in this section. The areas with height difference greater than ±1 m are highlighted with red marks (z≥1 m) and blue marks (z≤−1 m).

The density maps in Figure 15b,d appear similar, consistent with the performance indices of these two methods. On the other hand, Figure 15c manifests an excessive number of high-risk marks.

### 3.4. Comparison with State-of-the-Art Techniques

In [58], a satellite-based InSAR method utilizing a Kalman filter (KF) and sequential least squares (SLS) was introduced to implement near-real-time applications. The SLS was designed to reduce the CPU time of conventional LS methods by sequentially processing the whole image. For comparison, the results in Figure 7, Figure 10, Figure 12, Figure 14 and Figure 15 demonstrate the efficacy of the LSPU method, which incorporates 2D FFT in the LS method to reduce the CPU time even more significantly.

In [59], a deep learning-based LSPU method utilizing encoder–decoder architecture (PGENet) was proposed to reconstruct the wrapped phase data embedding noise. Similarly, a deep learning-based QGPU via global attention U-Net was introduced in [60]. The efficacy of LSPU and QGPU can be enhanced by utilizing a deep learning approach. Furthermore, the results in [59] demonstrated that LSPU outperformed QGPU, producing lower RMSE and shorter computational time, especially in low-coherence areas. The results in Figure 7c,e and Figure 12c,e show that the LSPU has nearly the same performance as the QGPU, not to mention that the LSPU has high computational efficiency, as listed in Table 2.

In [61], a weighted least-squares (WLS) technique was proposed to improve the effectiveness of phase unwrapping within a small baseline InSAR framework. Choosing a small baseline in a satellite-based InSAR approach can reduce the computational cost. The proposed UAV-based InSAR approach has relatively smaller (temporal and spatial) baseline compared to the satellite-based counterpart in [61]. In addition, the UAV-based platform offers more flexibility in achieving specific baseline and revisit time.

In [15], the low-coherence area and high-coherence area were filtered by a local fringe frequency compensation nonlocal filter and Goldstein filter, respectively. The Goldstein filter, considered an old-fashioned method, was used for its computational efficiency [15]. For the same reason, the mean filter adopted in our work is suitable for relatively smooth and high-coherence areas. In our approach, the data can be acquired with two UAVs (sensors) in a single flight or with one UAV (sensor) in two separate flights that are staggered by a short revisit time. The coherence in the UAV-based InSAR image pair is higher than that in the satellite-based counterpart, which has typical revisit time of 12 days or longer.

In [53], various filters were simulated upon ramp and square noisy images. The results indicated that the nonlocal filter outperformed both the Lee filter and the Goldstein filter (considered old-fashioned filters) on square noisy images, but underperformed the latter on ramp noisy images [53]. Such outcomes are consistent with the simulation findings presented in Section 3.1, Section 3.2 and Section 3.3. The scenarios simulated in Section 3.1 and Section 3.3 manifest relatively smooth height profiles, resembling ramp noisy images. Figure 7c,d and Figure 12c,d show that the mean filter achieves lower RMSE and higher SSIM value in these two scenarios. On the other hand, the scenario simulated in Section 3.2 manifests a steep mountain terrain, resembling square noisy images. Figure 10c,d show that nonlocal filter achieves lower RMSE in this scenario.

In the presence of additive Gaussian noise, the pivoting mean filter emerges as statistically optimal from the perspective of maximum likelihood estimation [20]. As for the scenarios with relatively smooth profile discussed in Section 3.1 and Section 3.3, reconstruction with mean filter (MF) results in slightly higher SSIM value and lower RMSE value compared with the nonlocal filter (NF). However, the mean filter may oversmooth the phase details in areas with drastic topographical variations. As discussed in Section 3.2, the scenario containing some steep areas may not be well reconstructed by using the mean filter, and the nonlocal filter achieves a lower RMSE on the reconstructed DEM.

In [62], a coherence-guided InSAR phase unwrapping method was proposed in conjunction with cycle-consistent adversarial networks. The coherence-guided phase unwrapping method typically employs a cost function in terms of phase gradients and coherence values to penalize phase discontinuities in low-coherence regions and promote smooth phase paths in high-coherence areas. The method could achieve accurate phase unwrapping with low RMS value. However, the generative adversarial networks entail high computational cost and require extensive training data.

In [63], a median filter was cascaded with a mean filter based on stationary wavelet transform for phase filtering. The median filter exceled in preserving phase fringes, while the mean filter demonstrated superior noise reduction capabilities.

Lightweight UAVs are typically more susceptible to wind disturbances than airborne platforms in conducting SAR or InSAR imaging tasks. Both types of platform may tilt or dip under headwinds and deviate from planned flight path under crosswinds [64]. Take a real-world example of dispatching a small UAV for InSAR imaging. It can carry a payload up to 7.5 kg and stay in the air for an hour while equipped with GPS navigation gear. Its attitude response to the wind interference can be ignored if the wind speed if less than 5 mph, and its trajectory deviation can be compensated with servo mechanisms and algorithms.

### 3.5. Discussions on Contributions and Constraints

The contributions of this work are summarized as follows:

1 An on-site InSAR imaging method is proposed for monitoring environmental changes. The imaging task is carried out with UAVs, which can be swiftly deployed on site with small decorrelation between master and slave images;
2 High-resolution DEMs are reconstructed and enhanced with a mean filter to mitigate artifacts on InSAR images, which are attributed to imperfect coregistration between master and slave images. A least-squares phase unwrapping method at extremely low computational cost is applied to run the imaging task near real-time;
3 Three scenarios of DEM reconstruction are simulated to validate the efficacy of the proposed approach, considering the effect of noise. The fidelity of acquired InSAR images is evaluated in terms of SSIM index and RMSE. The merits of using mean filter and least-squares phase unwrapping method are compared with two popular counterparts.

We propose a feasible scheme of deploying UAVs for on-site InSAR imaging of small areas, which cannot be achieved with satellite-borne InSAR platforms. Potential applications include monitoring natural disasters such as landslides, wildfires, and volcanic eruptions. In these scenarios, the satellite-borne InSAR imaging technique is limited by the long revisit time of days, which is impractical for real-time monitoring of disaster evolution. Among many state-of-the-art algorithms, choosing the mean filter and the least-square phase unwrapping method via Poisson’s difference equation and FFT can practically accomplish real-time imaging tasks in terms of robustness and computational efficiency.

The attitude of an airborne platform can be disturbed by complicated airflow disturbance and platform mechanical oscillation. Their effects on SAR imaging have been compensated with a compressive-sensing technique [46].

## 4. Conclusions

An on-site UAV-borne InSAR imaging method is proposed to reconstruct terrain profile with high spatial resolution in real time. A UAV-borne imaging system can be swiftly deployed to monitor rapidly changing environments during extreme weather events or natural disasters. Three different high-resolution DEMs are extracted from the USGS 3DEP datasets to validate the efficacy of the proposed approach. The combination of the least-squares phase unwrapping method featuring short CPU time and the mean filter for mitigating speckles on the acquired InSAR image is effective for monitoring terrain profile in real time. Several state-of-the-art techniques like nonlocal filter and quality-guided phase unwrapping method have been used to validate the advantages of the proposed approach to acquire InSAR images for reconstructing terrain profile in real time.

## Figures and Tables

**Table 1:** Default parameters for InSAR simulations.

Parameter	Symbol	Value
carrier frequency	$f_{c}$	1258 MHz
range bandwidth	$B_{r}$	300 MHz
pulse duration	$T_{r}$	1 μs
range sampling rate	$F_{r}$	360 MHz
range chirp rate	$K_{r}$	300 THz/s
range samples	$N_{r}$	1024
pulse repetition frequency	$F_{a}$	400 Hz
azimuth samples (case 1)	$N_{a}$	2048
azimuth samples (cases 2,3)	$N_{a}$	1024
look angle	$\theta_{\ell }$	$45^{\circ }$
platform height	*H*	2000 m
platform velocity	$v_{p}$	150 m/s
closest slant range	$R_{0}$	2828 m
slant range resolution	Δr	0.5 m
azimuth resolution (case 1)	Δa	0.44 m
azimuth resolution (cases 2,3)	Δa	0.88 m
baseline	*B*	5 m
height of ambiguity	$z_{\mathrm{amb}}$	80.52 m

**Table 2:** CPU time of running for LSPU, QGPU, mean filter, and nonlocal filter.

Method	CPU Time (s)
mean filter (MF)	962.80
nonlocal filter (NF)	1970.78
LS phase unwrapping	0.72
QG phase unwrapping	10,271.32

**Table 3:** RMSE and SSIM indices of acquired images under noise-free condition.

Figure 7	MF + LSPU	NF + LSPU	MF + QGPU
RMSE (m)	5.79	6.14	5.79
SSIM	0.90	0.89	0.90
Figure 10	MF + LSPU	NF + LSPU	MF + QGPU
RMSE (m)	28.40	28.24	24.91
SSIM	0.88	0.87	0.88
Figure 12	MF + LSPU	NF + LSPU	MF + QGPU
RMSE (m)	2.32	2.46	2.32
SSIM	0.90	0.89	0.90

**Table 4:** RMSE and SSIM indices of acquired images with proposed method under different SNRs.

Figure 7	SNR =0 dB	SNR =−5 dB	SNR =−10 dB
RMSE (m)	10.80	9.22	22.38
SSIM	0.89	0.89	0.74
Figure 10	SNR =0 dB	SNR =−5 dB	SNR =−10 dB
RMSE (m)	31.93	30.24	33.24
SSIM	0.87	0.86	0.78
Figure 12	SNR =0 dB	SNR =−5 dB	SNR =−10 dB
RMSE (m)	2.32	7.74	9.09
SSIM	0.90	0.72	0.66

## Data Availability

Publicly available datasets were analyzed in this study.

## Appendix A. Breakdown of CPU Time and Algorithms

Table A1 lists the number of multiplication/division operations in QGPU and LSPU, where $N_{a}$ and $N_{r}$ are the numbers of azimuth samples and range samples, respectively. The target area has the size of *U* azimuth cells by *V* range cells and is centered at sample indices $\left[N_{a}/2,N_{r}/2\right]$.

Algorithm A1 lists the procedure of the QGPU algorithm. The image sizes in Section 3.1, Section 3.2 and Section 3.3 are U×V=1401×841, 781×499, and 781×499, respectively. The average CPU time to run a complete cycle of phase unwrapping steps within the while loop (for one pixel) is about 0.01 s. For example, the image in Section 3.1 takes a CPU time of about 1401×841×0.01=11,782.41 s.

**Table A1:** Number of multiplication/division operations in QGPU and LSPU.

QGPU		LSPU	
**Process**	**No. of M/D**	**Process**	**No. of M/D**
$\Delta \phi_{u}\left[u^{\prime },v^{\prime }\right]$	none	${\bar{\bar{\phi }}}^{\left(4^{\prime }\right)}$	none
$\langle \Delta \phi_{u}\left[u,v\right]\rangle$	none	$\rho [u^{\prime },v^{\prime }]$	none
${\left(\Delta \phi_{u}\left[u^{\prime },v^{\prime }\right]-\langle \Delta \phi_{u}\left[u,v\right]\rangle \right)}^{2}$	1 per pixel	2D DFT	$UV\log_{2}UV$
${\left(\Delta \phi_{v}\left[u^{\prime },v^{\prime }\right]-\langle \Delta \phi_{v}\left[u,v\right]\rangle \right)}^{2}$	1 per pixel	Φ[m,n]	4UV
$Z{\left[u,v\right]}_{\left[u^{\prime },v^{\prime }\right]\in W_{s}}$	$2W_{s}$ per window	2D IDFT	$UV\log_{2}UV$
		$\phi^{\left(5\right)}\left[u^{\prime },v^{\prime }\right]$	none
total	2UV	total	$2UV(\log_{2}UV+2)$

**Algorithm A1:** Pseudocode of QGPU algorithm.

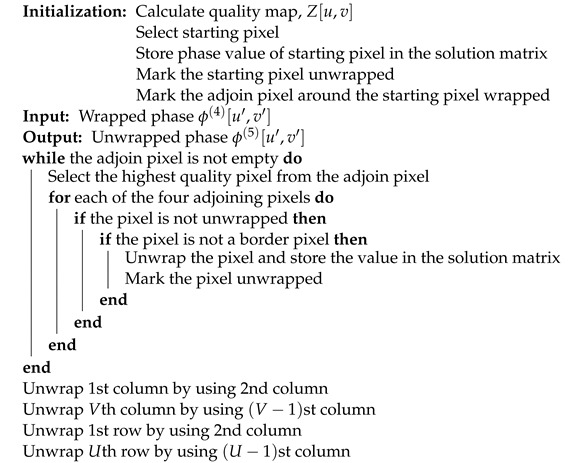

Table A2 lists the number in multiplication/division operations of nonlocal filter and mean filter. The total numbers of multiplication/division operations to run the nonlocal filter and the mean filter over an image of size U×V are 2UV and UV, respectively.

**Table A2:** Number of multiplication/division operations in nonlocal filter and mean filter.

Nonlocal Filter		Mean Filter	
**Process**	**No. of M/D**	**Process**	**No. of M/D**
$W_{1}\left[u,v;u^{\prime },v^{\prime }\right]$	1 per pixel	${\bar{\bar{\phi }}}^{\left(2\right)}$	none
$\phi_{\mathrm{NL}}^{\left(2\right)}\left[u,v\right]$	1 per pixel	${\bar{\bar{\phi }}}_{u^{\prime }v^{\prime }}^{\left(3\right)}$	none
		$A_{\phi_{s}}\left[u^{\prime },v^{\prime }\right]e^{j\phi_{s}\left[u^{\prime },v^{\prime }\right]}$	none
		$\phi^{\left(3^{\prime }\right)}\left[u^{\prime },v^{\prime }\right]$	1 per pixel
total	2UV	total	UV

Algorithm A2 lists the procedure for applying the mean filter on an image of size U×V. Algorithm A3 lists the procedure for applying the nonlocal filter on an image of size U×V. The average CPU time to run a complete cycle of mean filter on one pixel is about 0.005 s, and its counterpart with nonlocal filter is about 0.013 s.

**Algorithm A2:** Pseudocode of mean filter.

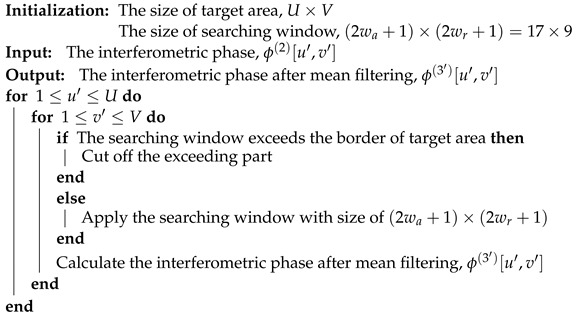

**Algorithm A3:** Pseudocode of nonlocal filter.
